# Monitoring ferroptosis *in vivo*: Iron-driven volatile oxidized lipids as breath biomarkers

**DOI:** 10.1016/j.redox.2025.103858

**Published:** 2025-09-02

**Authors:** Yuta Matsuoka, Yoshinori Katsumata, Po-sung Chu, Rei Morikawa, Nobuhiro Nakamoto, Kohta Iguchi, Ken Takahashi, Tadayuki Kou, Ryo Ito, Kojiro Taura, Shujiro Yazumi, Hiroaki Terajima, Gen Honjo, Genki Ichihara, Yuki Muramoto, Kazuki Sato, Rae Maeda, Kazuhiro Hata, Naoya Toriu, Motoko Yanagita, Masaki Tajima, Sidonia Fagarasan, Ken-ichi Yamada, Yuki Sugiura

**Affiliations:** a Multi-Omics Platform, Center for Cancer Immunotherapy and Immunobiology, Kyoto University Graduate School of Medicine, Kyoto, Japan; b Institute for Integrated Sports Medicine, Keio University School of Medicine, Tokyo, Japan; c Division of Gastroenterology and Hepatology, Department of Internal Medicine, Keio University School of Medicine, Tokyo, Japan; d Department of Gastroenterological Surgery and Oncology, Tazuke Kofukai Medical Research Institute, Kitano Hospital, Osaka, Japan; e Department of Gastroenterology, Tazuke Kofukai Medical Research Institute, Kitano Hospital, Osaka, Japan; f Department of Gastroenterology and Hepatology, Kyoto University Graduate School of Medicine, Kyoto, Japan; g Division of Cancer Immunotherapy, Center for Cancer Immunotherapy and Immunobiology, Kyoto University Graduate School of Medicine, Kyoto, Japan; h Department of Pathology, Tazuke Kofukai Medical Research Institute, Kitano Hospital, Osaka, Japan; i Tokai University, Graduate School of Medicine, Kanagawa, Japan; j Thermo Fisher Scientific K.K., Tokyo, Japan; k Department of Nephrology, Graduate School of Medicine, Kyoto University, Kyoto, Japan; l Institute for the Advanced Study of Human Biology, Kyoto University, Kyoto, Japan; m Division of Integrated High-Order Regulatory Systems, Center for Cancer Immunotherapy and Immunobiology, Kyoto University Graduate School of Medicine, Kyoto, Japan; n Laboratory for Mucosal Immunity, Center for Integrative Medical Sciences, RIKEN Yokohama Institute, Yokohama, Japan; o Department of Molecular Pathobiology, Faculty of Pharmaceutical Sciences, Kyushu University, Fukuoka, Japan; p Human Biology Microbiome Quantum Research Center, Keio University School of Medicine, Tokyo, Japan

**Keywords:** Ferroptosis, Breath analysis, Liver disease, Volatile oxidized lipid

## Abstract

Ferroptosis, an iron-dependent cell death mechanism characterized by excessive lipid peroxidation, has been implicated in numerous human diseases and organ pathologies. However, current detection methods necessitate invasive tissue sampling to assess lipid peroxidation, making noninvasive detection of ferroptosis in human subjects extremely challenging. In this study, we employed oxidative volatolomics to comprehensively characterize the volatile oxidized lipids (VOLs) produced during ferroptosis. Polyunsaturated fatty acid-derived VOLs were generated via iron-dependent LPO and released extracellularly as ferroptosis progressed. These VOLs were specifically generated during hepatic ferroptosis in mouse models of acetaminophen-induced liver injury and metabolic dysfunction-associated steatohepatitis (MASH) and were also detectable in the exhaled breath of patients with MASH. Specific VOLs released upon iron-dependent LPO are potential markers of ferroptosis *in vivo* and may facilitate noninvasive monitoring of cellular health in humans.

## Graphical abstract

**Figure:**
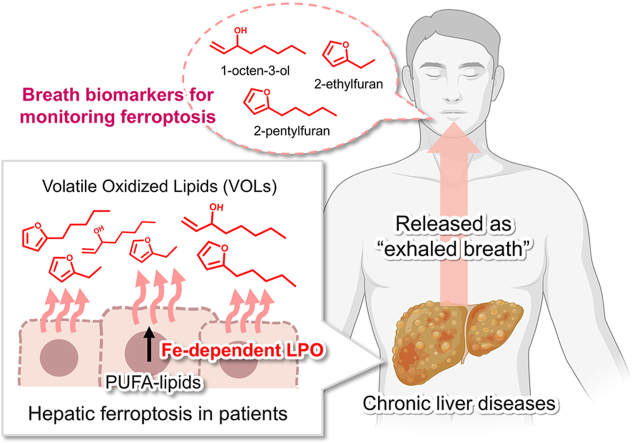

## 1 Introduction

Ferroptosis is a regulated cell death mechanism driven by iron-dependent lipid peroxidation (LPO) [1]. To date, ferroptosis has been implicated in a wide range of human diseases and organ pathologies [2], prompting numerous drug discovery studies targeting this process. To elucidate the role of ferroptosis in specific human diseases or pathological conditions, it is essential to detect LPO within damaged tissues [3]. Currently, the assessment of LPO in animal models and human tissues requires invasive tissue sampling followed by analysis using fluorescence probes or mass spectrometry. However, obtaining tissue samples from human subjects is frequently challenging, representing a major bottleneck in ferroptosis research.

During ferroptosis induction, LPO progresses within cellular membranes, leading to the fragmentation of membrane lipids. This produces volatile oxidized lipids (VOLs) that diffuse from cells and tissues, enter the circulation, and are released as part of exhaled breath [4]. Accelerated lipid fragmentation can result in plasma membrane rupture and ferroptosis [[5], [6], [7]]. Given that VOLs are generated alongside the progression of ferroptosis, they can serve as noninvasive ferroptosis markers. Hence, detecting ferroptosis-associated VOLs in exhaled breath may enable the noninvasive evaluation of disease progression.

The profile of VOLs produced during ferroptosis and in damaged tissues remains poorly defined, primarily due to the lack of established methodologies for elucidating the mechanisms responsible for small volatile molecule formation, which could be exploited to establish *in vitro* and *in vivo* measurement systems. Therefore, despite the considerable potential of VOLs as noninvasive biomarkers, there has been minimal effort to comprehensively analyze the chemical structures of VOLs produced by ferroptotic cells and damaged tissue.

To address this research gap, we performed oxidative volatolomics for a high-throughput analysis of VOLs released during ferroptosis. Three VOLs released from cells undergoing iron-dependent LPO were identified and confirmed as biomarkers for monitoring ferroptosis in liver disease. Notably, the levels of these markers were also higher in the breath of patients with metabolic dysfunction-associated steatotic liver disease (MASLD) than in healthy individuals, highlighting their potential as noninvasive markers for disease monitoring.

These molecules serve as noninvasive markers for evaluating iron-dependent LPO, a key hallmark of ferroptosis [3], at the human level, and are valuable for monitoring pathological conditions associated with ferroptosis.

## 2 Materials and methods

### 2.1 Materials

Linoleic acid (FA18:2), arachidonic acid (FA20:4), eicosapentaenoic acid (FA20:5), and docosahexaenoic acid (FA22:6) were purchased from Nacalai Tesque, Inc. (Kyoto, Japan). 2,2′-Azobis (2-methylpropionamidine) dihydrochloride (AAPH) and *N*-acetyl cysteine (NAC) were obtained from Wako Pure Chemical Industries (Osaka, Japan), and hemin was procured from Tokyo Chemical Industry Co., Ltd. (Tokyo, Japan), and acetaminophen (APAP) was purchased from Sigma-Aldrich (St. Louis, MO, USA). (1S,3R)-RSL3, FINO2, ML162, liproxstatin-1, staurosporine, 15-HpETE, 12-HpETE, 9-HpODE, 13-HpODE, arachidonic acid-d5 (FA20:4-d5), linoleic acid-d4 (FA18:2-d4), arachidonic acid-d8 (FA20:4-d8), and docosahexaenoic acid-d5 (FA22:6-d5)were obtained from Cayman Chemical (Ann Arbor, MI, USA). Acetonitrile (LCMS grade, ≥99.9 %), isopropanol (LCMS grade, ≥99.9 %), methanol (LCMS grade, ≥99.9 %), water (LCMS grade, ≥99.9 %), and ammonium formate (Wako 1st grade) were purchased from Wako Pure Chemical Industries, Ltd.

### 2.2 Cell culture

HepG2, Calu-1, and HEK293 cells were purchased from American Type Culture Collection (Manassas, VA, USA). HepG2 and HEK293 cells were cultured in high-glucose Dulbecco's Modified Eagle Medium (DMEM), whereas Calu-1 cells were cultured in McCoy's 5A medium. All growth media were supplemented with 10 % fetal bovine serum (FBS) and 100 U/mL of penicillin and streptomycin. All cell lines were maintained at 37 °C in a humidified incubator with a 5 % CO_2_ atmosphere, passaged for <6 months, and were not further tested or authenticated by the authors.

### 2.3 Cytotoxicity assay

Cells (1 × 10^4^ cells/well) were plated in 96-well flat-bottom plates and cultured for 24 h before exposure to various concentrations of compounds at 37 °C. The amount of LDH in the supernatant was measured using a cytotoxicity LDH assay kit-WST (Dojindo Molecular Technologies, Inc., Kumamoto, Japan) following the manufacturer's instructions. For the MTT assay, 15 μL of 5 mg/mL MTT solution (Nacalai Tesque, 23547-21) was added to each well and incubated at 37 °C for 1 h. The supernatant was removed, and 100 μL of dimethyl sulfoxide (DMSO) was added to each well. Absorbance was measured at 490 nm (LDH) and 570 nm (MTT assay) using a 96-well plate reader (Infinite F50; Tecan Trading AG, Switzerland).

### 2.4 Isotope labeling of VOLs generated during ferroptosis

Screening-1 (^18^O_2_/H_2_^18^O labeling): HepG2 cells (1 × 10^6^ cells) were seeded into culture flasks and incubated for 24 h. Subsequently, the culture medium was replaced with DMEM prepared using H_2_^18^O and supplemented with 10 % FBS. The flask was then hermetically sealed, and ^18^O_2_-enriched air (composition: N_2_ 74 %, ^18^O_2_ 21 %, and CO_2_ 5 %) generated using the CUBE GM-X3 (FCON CO., LTD., Kochi, Japan) was introduced into the flask at a flow rate of 50 mL/min for 20 min via a syringe, thereby replacing the internal atmosphere with ^18^O_2_ air. Subsequently, a ferroptosis inducer (with or without a ferroptosis inhibitor) was added, and after a defined incubation period, the headspace gas above the cell culture was collected. Sampling was conducted using the SP209-1000 Dual device (GL Sciences Inc., Tokyo, Japan) at a flow rate of 50 mL/min for 30 min, and the volatiles were trapped in inert-coated biomonitoring tubes (Markes International, Llantrisant, UK). The collected samples were subsequently analyzed using thermal desorption–gas chromatography/high-resolution mass spectrometry (TD-GC/HRMS).

Screening-2 (d-labeled PUFA labeling); HepG2 cells (5 × 10^5^ cells) were seeded into culture flasks and incubated for 24 h. Subsequently, d-labeled PUFAs (FA20:4-d5, FA18:2-d4, FA20:4-d8, and FA22:6-d5) were added at a final concentration of 20 μM, followed by additional 24-h incubation. After this treatment, the culture medium containing the D-labeled PUFAs was replaced with standard DMEM, and ferroptosis induction, gas collection, and analysis were performed in the same manner as described in Screening-1.

### 2.5 VOL analysis by TD-GC/HRMS

The TD injection system was a TD100-xr (Markes International, California, USA) equipped with Tenax TA focusing trap. The sample path was set to 230 °C, and the flow rate was adjusted to 10:1 in the split mode. The cold focusing trap was set to −30 °C, and trap desorption was performed at 280 °C. A TRACE 1610 Gas Chromatograph (Thermo Fisher Scientific, San Jose, CA, USA) equipped with a TG-5SILMS column (60 m, 0.25 mm ID, 0.25 μm FT, Thermo Fisher Scientific) was used. The carrier flow was set to 1 mL/min using helium. The ramped oven program was set as follows: 30 °C (5 min hold), 5 °C/min to 150 °C, 10 °C/min to 280 °C (20 min hold).

An Orbitrap Exploris GC 240 mass spectrometer (Thermo Fisher Scientific) was used. Data acquisition was performed in full scan mode (*m*/*z* 35–450) with a resolving power of 60,000 (FWHM) at *m*/*z* 200. HRMS data were analyzed using Compound Discoverer software (Thermo Fisher Scientific).

Unlabeled and labeled raw data were employed to distinguish between isotope-unlabeled and isotope-labeled mass spectra, and the retention times at which the labeled spectra were detected were extracted. Next, unlabeled raw data were employed to identify the components corresponding to the extracted retention times from the component table obtained through deconvolution, NIST spectrum search, high-resolution filtering, and the retention index.

**Fig. 1:**
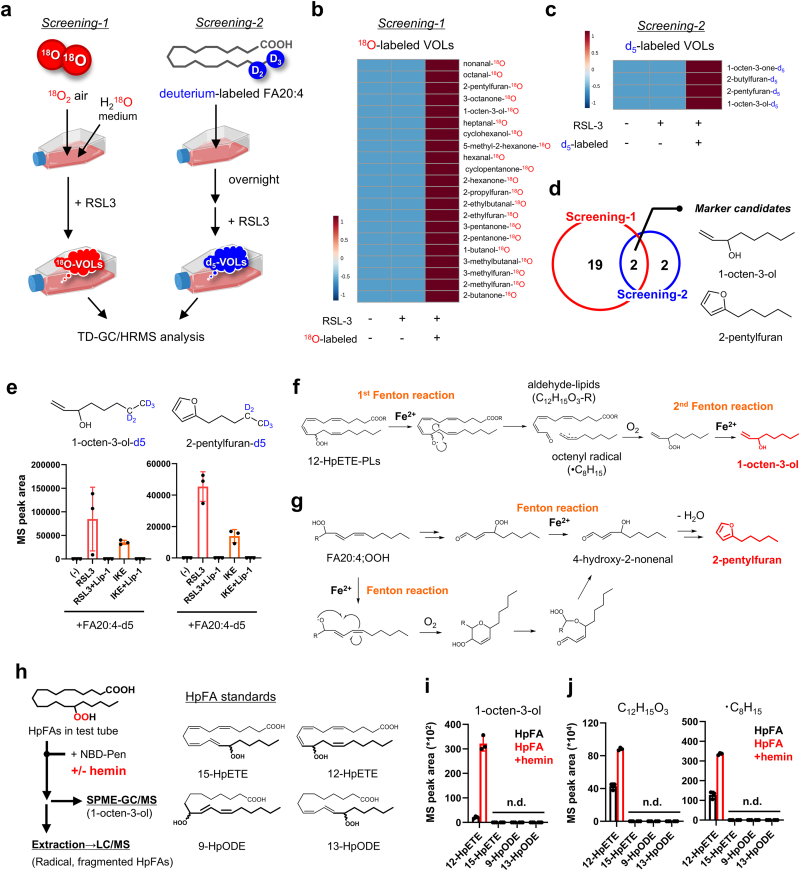
**Identification of volatile oxidized lipids (VOLs) released during ferroptosis using volatile oxylipidomics.** (a) Overview of volatile oxylipidomics. (b, c) Hierarchical cluster analysis of VOLs generated in each screening system. (d) Number of volatile organic compounds (VOC) hits identified in each screening and chemical structures of the commonly detected molecules (1-octen-3-ol and 2-pentylfuran). (e) HepG2 cells were supplemented with FA20:4-d5 to incorporate labeled fatty acids into cell membrane lipids and subsequently treated with 3.0 μM RSL3 or 20 μM IKE in the presence or absence of 1.0 μM Lip-1. After treatment, the amount of d5-labeled VOLs was quantified using thermal desorption–gas chromatography/high-resolution mass spectrometry (TD-GC/HRMS). (f, g) Plausible generation mechanisms of 1-octen-3-ol (f) and 2-pentylfuran (g). (h) Protocol for structural analysis of lipid fragments derived from hydroperoxy fatty acids (HpFAs) after addition of hemin and the chemical structures of the HpFAs used. (i, j) 1-octen-3-ol (i) and C12H15O3/•C8H15. black; HpFA only, red; HpFA + hemin. Data are presented as the mean ± standard deviation of three repeated experiments.

TD-GC/HRMS was employed for VOL analysis, as shown in Fig. 1b, c, and e.

### 2.6 Hydroperoxy fatty acid (HpFA)-derived VOLs, radicals and lipid fragments analysis

HpFA/hemin-induced oxidation was performed by incubating a solution containing 10 μM HpFAs, 10 μM hemin, and 10 μM NBD-Pen in PBS (pH 7.4) at 37 °C for 15 min. For the VOL analysis, the reaction was conducted in an SPME vial. After the designated reaction period, the volatile compounds were analyzed using SPME-GC/MS. For radical and lipid fragment analyses, the mixture was extracted using the modified Bligh and Dyer method. The extracted solution was dried under a nitrogen gas stream, and the residue was dissolved in methanol (200 μL) and stored at −80 °C before injection into the LC/HRMS system.

### 2.7 VOL analysis by SPME-GC/MS

The GC/MS system used was an Agilent 5977 B GC/MSD. An SPME autosampler was used as a PAL-RTC multifunctional sampler for gas chromatography. Chromatographic separation was achieved using an HP-5ms column (60 m × 0.25 mm, I.D. × 0.25 μm), with helium as the carrier gas at a flow rate of 1.5 mL/min. The injector temperature was maintained at 250 °C. The column oven temperature program began at 35 °C, was held for 4 min, increased to 150 °C at a rate of 5 °C/min, increased to 280 °C at a rate of 10 °C/min, and was finally held for 10 min. Electron ionization (EI) method employing scan (test-tube experiments) and SIM (cell culture experiments) analyses was used. GC/MS analysis was controlled using MassHunter software, with autosampler operations managed using PAL Sample Control software.

VOL extraction was performed using a SPME Arrow fiber (SARR15-DVB/CWR120/20-P 1). The vial was agitated at 40 °C and 500 rpm for 900 s, followed by the extraction of VOLs from the headspace for 600 s under the same conditions. Subsequently, the SPME arrow fiber was inserted into the GC/MS injector and desorbed at 250 °C for 60 s.

**Fig. 2:**
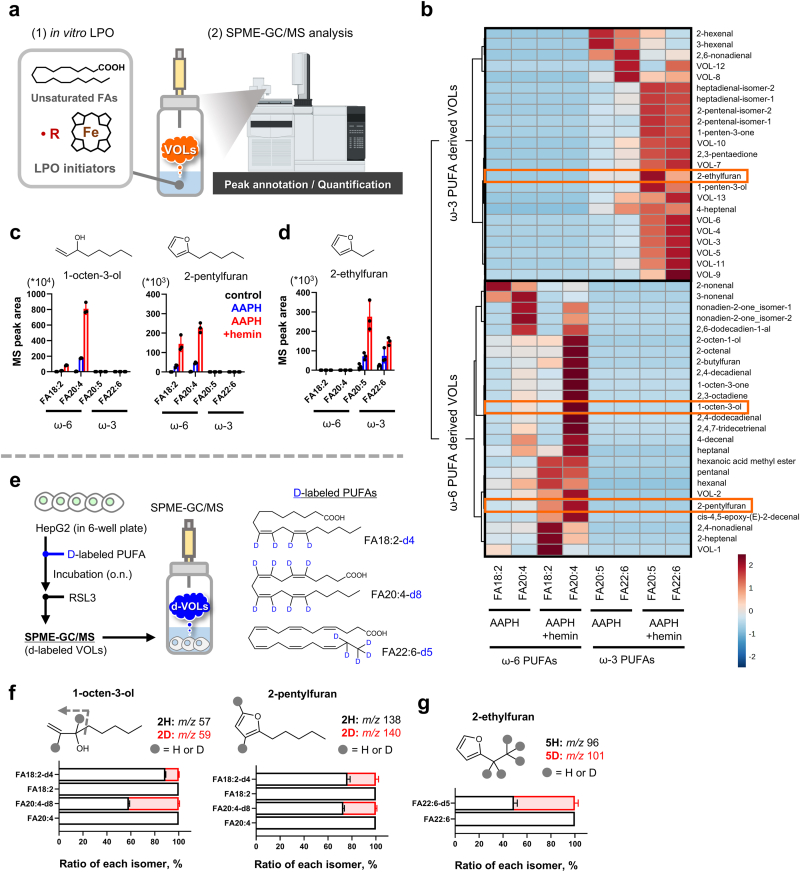
**1-octen-3-ol and 2-pentylfuran are produced from ω-6 PUFAs, and 2-ethylfuran from ω-3 PUFAs, through iron-dependent lipid peroxidation (LPO) during ferroptosis.** (a) Outline of the SPME-GC/MS-based identification of PUFA-derived VOLs generated by AAPH or AAPH + hemin stimulation. (b) Hierarchical cluster analysis of VOLs generated in each peroxidation system. Original data are shown in **Supplementary**Table 1. (c, d) MS peak areas of typical ω-6/ω-3-PUFA-derived VOLs observed in each LPO system. (c) 1-octen-3-ol/2-pentylfuran and (d) 2-ethylfuran. (e) Strategy for deuterium (d)-labeling of intracellular PUFA-containing phospholipids and VOLs and the chemical structures of the d-labeled PUFA used. HepG2 cells were treated with 20 μM d-labeled PUFAs for 24 h, followed by the addition of 3.0 μM RSL3. (f, g) Isotopomer abundances of 1-octen-3-ol, 2-pentylfuran (f), and 2-ethylfuran (g) after (d-labeled) PUFA and RSL3 treatment. The MS peak area of each VOL sample was calculated from the corresponding ion chromatogram. Data are presented as the mean ± standard deviation of three repeated experiments.

**Fig. 3:**
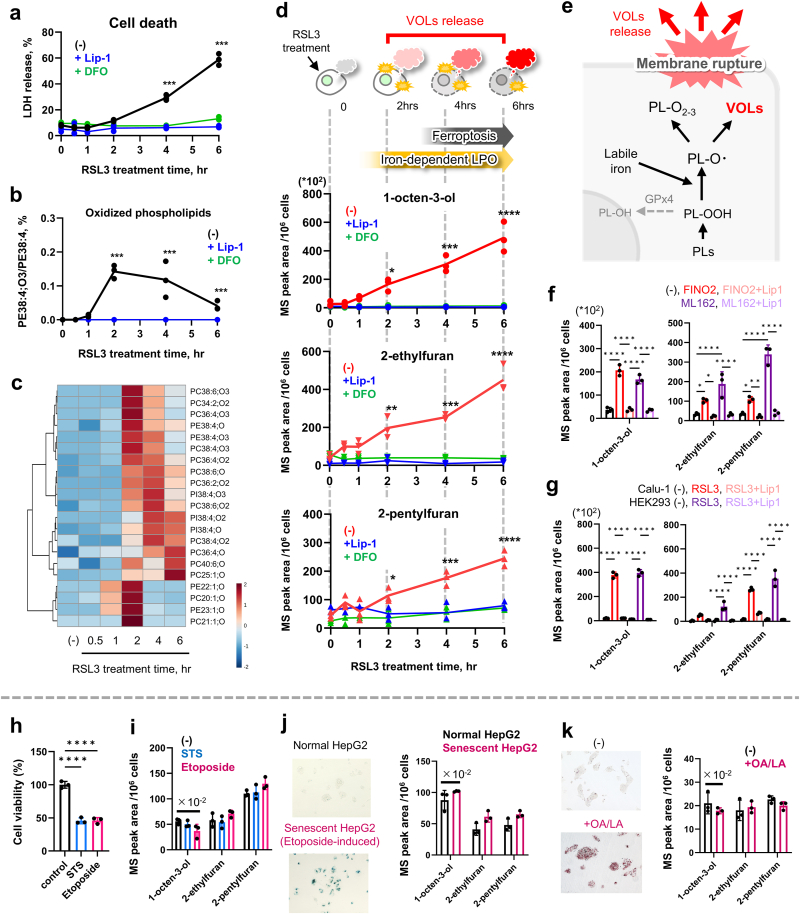
**Ferroptosis progression specifically leads to the release of 1-octen-3-**ol**, 2-pentylfuran, and 2-ethylfuran.** (a–d) Time-course analysis of LDH release (a), oxidized lipid production (b, c), and the levels of VOLs in the cellular headspace (d) after treating HepG2 cells with 3.0 μM RSL3. (e) Plausible mechanisms for VOL formation and release during ferroptosis. (f) Levels of 1-octen-3-ol, 2-pentylfuran and 2-ethylfuran after treating HepG2 cells for 4 h with other ferroptosis inducers (25 μM FINO2, 3.0 μM ML162). (g) Levels of 1-octen-3-ol, 2-pentylfuran. and 2-ethylfuran following ferroptosis induction in Calu-1 and HEK293 cells using 0.5 μM RSL3. (h, i) Levels of 1-octen-3-ol, 2-pentylfuran, and 2-ethylfuran after treating cells with apoptosis inducers (10 μM staurosporine [STS], 100 μM etoposide) for 6 h. (j, k) Release of VOLs from etoposide-treated HepG2 cells (j) or HepG2 cells with lipid accumulation induced by oleic acid/linoleic acid (k). Data are presented as the mean ± standard deviation of six independent experiments. Statistical significance was assessed using a one-way analysis of variance with Tukey's multiple comparison test. ∗*p* < 0.05, ∗∗*p* < 0.01, ∗∗∗*p* < 0.001, ∗∗∗∗*p* < 0.0001.

**Fig. 4:**
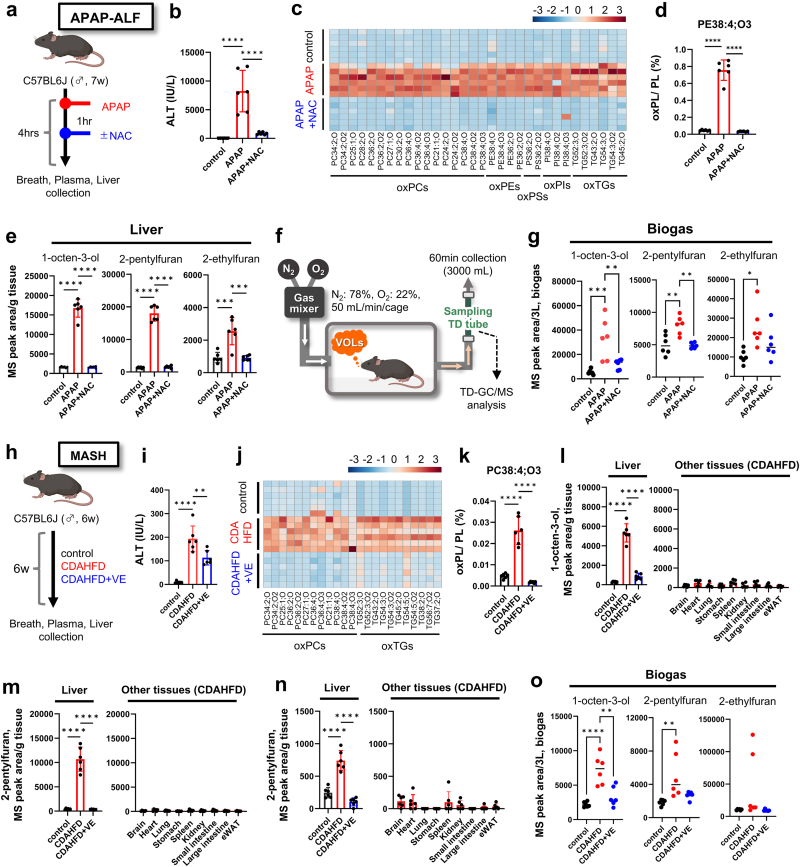
**Emission of ferroptosis VOL markers as biogas from acute or chronic liver disease model mice.** (a) Experimental procedure for VOL analysis in APAP-ALF model mice. (b–e) Plasma alanine aminotransferase (ALT) levels (b), hepatic oxidized lipids (c, d), and hepatic VOL levels (e) in control and 300 mg/kg APAP (±NAC)-treated groups. (f) Experimental setup for gas collection from mice. (g) Levels of 1-octen-3-ol, 2-pentylfuran, and 2-ethylfuran in biogas released from APAP-ALF model mice. (h) Experimental procedure for VOL analysis in choline-deficient, l-amino acid-defined, high-fat diet (CDAHFD)-fed metabolic dysfunction-associated steatohepatitis (MASH) model mice. (I–k) Plasma ALT levels (i) and hepatic oxidized lipid levels (j, k) in control and CDAHFD (±VE)-fed groups. (l–o) Levels of 1-octen-3-ol, 2-pentylfuran, and 2-ethylfuran in tissues (l–n) and biogas (o). Data are presented as the mean ± standard deviation of six independent experiments. Statistical significance was assessed using a one-way analysis of variance with Tukey's multiple comparison test. ∗*p* < 0.05, ∗∗*p* < 0.01, ∗∗∗*p* < 0.001, ∗∗∗∗*p* < 0.0001.

The identified VOLs were analyzed using MassHunter Workstation Qualitative Analysis software. Compounds were identified using the NIST MS Search ver. 2.4 and the NIST Mass Spectral Library. Mass spectra from the experiments were compared with those of the selected candidate compounds and were further verified by comparing the GC/MS results with those of the standard samples of these candidates. Peaks that were not identified in the library search were designated VOL-n (number). Additionally, peak area values were computed from the mass chromatograms of the most intense fragment peaks for each compound. SPME-GC/MS was employed for VOL analysis, as shown in Fig. 1, Fig. 2, Fig. 3, Fig. 4e, l-n, and Extended Data Figs. 4 and 5.

### 2.8 Oxidation of standard PUFAs using AAPH/hemin

In an SPME vial, PUFAs (500 μM), AAPH (50 mM), and hemin (10 μM) were mixed in phosphate-buffered saline (PBS; pH 7.4, containing 0.1 % ethanol). After sealing the vial, the reaction mixture was incubated at 37 °C for 2 h. The samples were analyzed using SPME-GC/MS analysis.

### 2.9 Collection and analysis of cellular VOLs using SPME-GC/MS

HepG2 cells were seeded (5 × 10^5^ cells/well) in 6-well culture plates and allowed to adhere for 24 h before treatment. Ferroptosis was induced using RSL3 (3.0 μM for HepG2, 0.5 μM for Calu-1, and HEK293), FINO2 (25 μM), and ML162 (3.0 μM). Apoptosis was induced by STS (10 μM) and etoposide (100 μM). Cells were also treated with the ferroptosis inhibitors, Lip-1 (1.0 μM) and DFO (100 μM). Subsequently, the cells and culture medium were transferred to an SPME vial and analyzed using SPME-GC/MS.

### 2.10 Lipid measurements using LC/HRMS

To extract lipids, 1000 μL of a 1:1 (v/v) 1-butanol/methanol solution containing 100 μM butylated hydroxytoluene (BHT) and 100 μM ethylenediaminetetraacetic acid (EDTA) was added to the cell pellets. The mixture was vortexed for 10 s, sonicated for 15 min in an ice-cooled sonic bath, and centrifuged at 16,000×*g* for 10 min at 20 °C. The supernatant was transferred into a 0.2 mL glass insert with a Teflon-lined cap for liquid chromatography electrospray ionization mass spectrometry (LC-ESI-MS) analysis.

For lipid analysis, a Q-Exactive Focus mass spectrometer (Thermo Fisher Scientific) was connected to an Ultimate 3000 HPLC system (Thermo Fisher Scientific). Samples were separated using a Thermo Scientific Accucore C18 column (2.1 × 150 mm, 2.6 μm) with mobile phase A comprising 10 mM ammonium formate in 50 % acetonitrile with 0.1 % formic acid and mobile phase B comprising 2 mM ammonium formate in a mixture of acetonitrile, isopropyl alcohol, and water (10:88:2, v/v/v) with 0.02 % formic acid. The step gradient was as follows: 65:35 (0 min), 40:60 (0–4 min), 15:85 (4–12 min), 0:100 (12–21 min), 0:100 (21–24 min), 65:35 (24–24.1 min), and 100:0 (24.1–28 min), at a flow rate of 0.4 mL/min, and a column temperature of 35 °C.

The Q-Exactive Focus mass spectrometer was operated in the ESI positive and negative ion modes. The ion source parameters comprised a spray voltage of 3 kV, transfer tube temperature of 285 °C, S-Lens level of 45, heater temperature of 370 °C, sheath gas flow rate of 60, and auxiliary gas flow rate of 20. LC/HRMS in the positive and negative ion modes was employed for the relative quantification of oxidized lipids based on previously published methods [38,39]. The experimental conditions for full-scan MS were as follows: resolving power = 70,000, automatic gain control target = 1 × 10^6^, trap fill time = 100 ms, and scan range = *m*/*z* 250–1500. The precursor and product ions of the d-labeled phospholipids analyzed by PRM are listed in Supplementary Table 3. The experimental conditions for PRM were as follows: resolving power = 17,500, automatic gain control target = 1 × 10^6^, and trap fill time = 100 ms. The acquired data were analyzed using Qual Browser (Thermo Fisher Scientific).

### 2.11 Apoptosis assay

HepG2 cells (5 × 10^5^ cells/well) were seeded in a six-well plate and cultured for 24 h before treatment. After treating the cells with 10 μM STS or 100 μM etoposide for 6 h, the cells were washed with PBS. Accutase (Nacalai Tesque, Inc. Kyoto, Japan) was used to detach cells, which were then washed with 1 mL HBSS. To detect apoptosis, the Annexin V-633 Apoptosis Detection Kit (Nacalai Tesque, Kyoto, Japan) was used according to the manufacturer's protocol. Cells were passed through a cell strainer and assayed using a flow cytometer (BD FACSCanto™ II, BD Biosciences, Franklin Lakes, NJ, USA) with 488 nm/633 nm laser for excitation. Fluorescence intensity was measured using a 660/20 band-pass filter for Annexin V-APC and a 575/26 band-pass filter for propidium iodide. At least 10,000 events were collected for each sample. Data were analyzed using FlowJo software (BD Biosciences).

### 2.12 Senescence-associated β-galactosidase staining

HepG2 cells (1 × 10^5^ cells/well) were seeded in 35-mm dishes and cultured for 7 days in DMEM supplemented with 2.0 μM etoposide. Senescence-associated β-galactosidase (SA-β-Gal) activity was detected using a Senescence β-Galactosidase Staining Kit (Cell Signaling Technology) according to the manufacturer's instructions. Stained cells were observed using a fluorescence microscope (BZ-8000; Keyence, Osaka, Japan).

### 2.13 Oil Red O staining

HepG2 cells (1 × 10^5^ cells/well) were seeded in 35-mm dishes and cultured for 2 days in DMEM supplemented with a fatty acid–albumin complex (linoleic acid and oleic acid in a 2:1 M ratio, Sigma-Aldrich). Lipid accumulation was quantified using an Oil Red O solution (0.5 % v/v). Cell images were captured using a BZ-8000 fluorescence microscope (BZ-8000).

### 2.14 Animal model

Male C57BL/6J mice were acquired from CLEA Japan, Inc. (Tokyo, Japan). All mice were housed under sterile conditions at a temperature of 24 ± 1 °C, humidity of 60 ± 10 %, and a 12-h light-dark cycle. Drinking water and food were provided *ad libitum* during the study period. The animal study was approved by the Animal Studies Committee of Kyoto University.

APAP-acute liver failure (ALF) model mice: A single dose of APAP (300 mg/kg body weight) was intraperitoneally administered to overnight-fasted mice aged eight weeks. Six mice were used in each of the experimental groups. To suppress APAP-induced LPO, NAC (300 mg/kg body weight) in saline was intraperitoneally injected 1 h after APAP administration.

MASH model mice: Male mice aged 6 weeks were randomly divided into three groups fed a standard diet (SD), a choline-deficient, l-amino acid-defined, high-fat diet with 60 kcal% fat and 0.1 % methionine without added choline (CDAHFD; Research Diets A06071302, Research Diets, Inc. New Brunswick, NJ, USA) or CDHFD with vitamin E (1.0 g/kg diet) for six weeks to establish a MASH mouse model. Six mice were used in each experimental group.

### 2.15 VOL analysis using TD-GC/MS

**Fig. 5:**
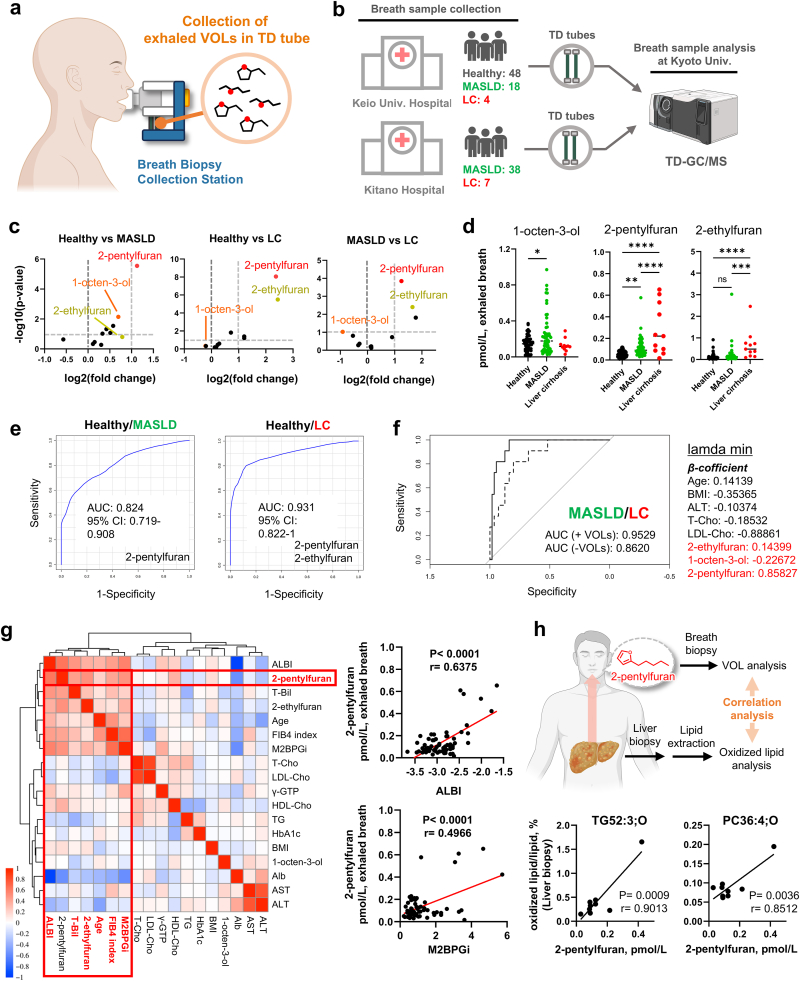
**Elevated exhaled levels of ferroptosis VOL markers in patients with metabolic dysfunction-associated steatotic liver disease/liver cirrhosis (MASLD/LC) compared with healthy individuals.** (a) Collection of exhaled VOLs using the Breath Biopsy Collection Station. (b) Implementation framework for clinical trials. (c) Volcano plots depicting the distribution of VOLs in breath samples from patients with each disease condition. (d) Semi-quantification of ferroptosis VOL markers in the exhaled breath of each group. (e) ROC curve analysis of the VOLs of healthy vs MASLD (2-pentylfuran) and healthy vs LC (2-pentylfuran and 2-ethylfuran) comparisons. (f) ROC curve analysis using a combination of VOLs and clinical markers of the MASLD vs LC comparison. Solid line: ROC curve analysis for combined clinical markers and VOLs (AUC; 0.9529); dashed line: ROC curve analysis for clinical markers only (AUC; 0.8620). (g) Correlation analysis between ferroptosis VOL markers and clinical markers. ALBI; Albumin-bilirubin score, T-Bil; Total bilirubin, M2BPGi; Mac-2 binding protein glycosylation isomer, T-Cho; Total cholesterol, LDL-Cho; Low-density lipoprotein cholesterol, HDL-Cho; High-density lipoprotein cholesterol, TG; Triglyceride, Alb; Albumin.　For the 2-pentylfuran and M2BPGi and ALBI correlation, two-sided Pearson's correlation (*r*) and *p*-values are indicated. (h) Correlation between oxidized lipid levels in liver biopsy samples and 2-pentylfuran level in exhaled breath. Statistical significance (d) was assessed using one-way analysis of variance with Tukey's multiple comparison test. ∗*p* < 0.05, ∗∗*p* < 0.01, ∗∗∗*p* < 0.001, ∗∗∗∗*p* < 0.0001.

A Shimadzu GCMS-QP2020NX thermal desorption system (Shimadzu TD-30R, Shimadzu Corporation, Kyoto, Japan) was used for the GC/MS analysis. The separation column employed was an HP-5ms (60 m × 0.25 mm, I.D. × 0.25 μm). Helium was used as the carrier gas at a flow rate of 1.5 mL/min. The injector temperature was maintained at 250 °C. The temperature program for the column oven started at 35 °C, was held for 4 min, increased to 150 °C at 5 °C/min, increased to 280 °C at 10 °C/min, and was held for 10 min. EI was employed for ionization, and selected ion monitoring (SIM) analysis was performed. Volatile oxidized lipids were analyzed using the Shimadzu LabSolutions software (Shimadzu Corporation, Kyoto, Japan). TD-GC/MS was employed for VOL analysis, as shown in Fig. 4, Fig. 5.

### 2.16 Collection of VOLs in mouse-derived gas

Mice were placed in a sealed chamber into which artificial air (N_2_:78 %, O_2_:22 %) generated using CUBE GM-X3 was introduced at a flow rate of 50 mL/min. An inert-coated biomonitoring tube was connected to the outlet of the gas line, and gas sampling was performed for 60 min. The collected gas samples were analyzed using TD-GC/MS.

### 2.17 Extraction of VOLs and oxidized lipids from mouse tissues

VOLs were extracted from mouse tissues using an extraction solution (methanol containing 100 μM dibutyl hydroxytoluene [BHT] and 100 μM EDTA). Briefly, 1 mL of extraction solution was added to 50 mg of frozen tissue and homogenized using a Bertin Minilys homogenizer (Bertin Technologies SAS, Montigny-le-Bretonneux, France). The homogenate was sonicated for 5 min on ice, allowed to stand for 5 min, and centrifuged (6000×*g* for 10 min at 4 °C). The supernatant (∼700 μL) was collected, and the extracted samples were stored at −80 °C until measurement.

### 2.18 Histopathology

Mouse liver was freshly sampled, snap-frozen in liquid nitrogen, embedded in SCEM compound (Leica Microsystems, Tokyo, Japan), and stored at −80 °C until analysis. After freezing, 6-μm-thick cryostat sections were obtained at −12 °C. Frozen sections were stained with hematoxylin and eosin (H&E). Images were obtained using a slide scanner (Fino CLARO; DRC, Tokyo, Japan).

### 2.19 Human exhaled breath sample collection

The trial protocol was approved by the ethics committees of Keio University Hospital (approval no. 20221057) and Kitano Hospital (approval no. P221100201). All procedures were conducted according to the Declaration of Helsinki and the Ethical Guidelines for Medical and Health Research Involving Human Subjects (Japanese Ministry of Health, Labour and Welfare). Informed consent was obtained from all the participants.

Forty-eight healthy participants and 67 patients diagnosed with MASLD or liver cirrhosis (LC) were enrolled between February 2023 and February 2025 at Keio University Hospital and Kitano Hospital (Supplementary Table 2). Healthy individuals had no comorbidities, such as hypertension, diabetes, or active lung disease. Patients who had difficulty using a mask for 15 min or required oxygen therapy were excluded from the study. All enrolled patients fulfilled the diagnostic criteria for nonalcoholic fatty liver disease (NAFLD) [40] and MASLD [41], with MASH diagnosed according to the Delphi consensus statement [41], and LC diagnosed according to the most recent Japanese Guideline [42]. The MASLD group included 56 patients (including those with MASH but without cirrhosis), and the LC group included 11 patients. The median ages were 58.0 years (interquartile range [IQR] 32–87) and 69.6 years (IQR 58–81), respectively, with female proportions of 60.7 % and 54.5 %. All LC cases were categorized as Child–Pugh class A. The albumin-bilirubin (ALBI) scores were −2.98 (IQR −3.67 to −2.33) for MASLD and −2.43 (IQR −3.33 to −1.65) for LC; the FIB-4 indices were 1.73 (IQR 0.30–6.61) for MASLD and 4.78 (IQR 1.77–10.86) for LC; and M2BPGi levels were 0.99 (IQR 0.21–3.3) and 2.85 (IQR 0.54–5.73), respectively.

Breath samples were collected at each institution using breath biopsy collection stations and stored in thermal desorption tubes (Fig. 5a). The tubes were transported to Kyoto University for TD-GC/MS analysis (Fig. 5b). This multicenter approach enabled the consistent collection and measurement of exhaled breath from geographically distinct sites, ensuring broad applicability.

### 2.20 Breath collection using the breath biopsy collection station

Breath samples were obtained using inert-coated biomonitoring tubes connected to a ReCIVA Breath Sampler (Owlstone Medical, Cambridge, UK). Prior to sample collection, the tubes were prepared in a TD-30R (Shimadzu) under a 20-psi nitrogen gas flow at 280 °C for 30 min. Before sampling, the system was calibrated and adjusted according to each individual's breathing pattern. Each tube collected approximately 1.0 L of breath at a flow rate of 200 mL/min. A CASPER Portable Air Supply (Owlstone Medical, Cambridge, UK) was used to minimize ambient contamination. The tubes were stored at −20 °C, and VOL analysis was performed within one week of collection.

### 2.21 Liver biopsy

Among the patients enrolled in this study, those scheduled to undergo liver resection for liver tumors underwent sharp dissection biopsy of non-tumor background liver tissue from the planned resection area during surgery. Liver biopsy specimens were immediately snap-frozen in liquid nitrogen and stored at −80 °C.

### 2.22 Statistical analyses

Statistical analyses were conducted using GraphPad Prism (version 9.2.0, GraphPad Software, Inc., San Diego, CA, USA), and data were analyzed using unpaired Student's *t*-test, one-way analysis of variance (ANOVA) with Tukey's multiple comparisons test, two-sided Pearson's correlation, or two-way ANOVA with Tukey's multiple comparisons tests. The results are expressed as mean ± standard deviation (SD). Statistical significance was set at *p* < 0.05. LASSO regression analysis was performed using the Glmnet R package (v4.1–8) and R v4.3.2. Multivariate ROC curves and AUCs were derived using the “glm” function in R (version 4.3.2).

## 3 Results

### 3.1 Ferroptosis-derived volatile markers: 1-octen-3-ol and 2-pentylfuran

LPO during ferroptosis is characterized by (1) the formation of lipid hydroperoxides following the addition of molecular oxygen (O_2_) to polyunsaturated fatty acid-containing phospholipids (PUFA-PLs) and (2) the subsequent generation of highly oxidized lipids—including truncated short-chain molecules—via Fenton reactions between lipid hydroperoxides and labile iron. We hypothesized that comprehensive profiling of VOLs released from peroxidized PUFAs would facilitate the identification of ferroptosis-associated VOL markers.

To test this hypothesis and minimize confounding factors from non-cellular sources (e.g., culture medium or environmental contaminants), two complementary isotope-tracing approaches were employed using ^18^O_2_ and deuterium-labeled arachidonic acid (FA20:4-d_5_). Briefly, HepG2 cells were treated separately with ^18^O_2_ (Screening-1) or FA20:4-d_5_ (Screening-2) before ferroptosis induction. Oxidative volatolomics was then used to profile the isotope-labeled VOLs released into the gas phase (Fig. 1a).

In Screening-1, ferroptosis was induced in HepG2 cells cultured in an ^18^O_2_ air/H_2_^18^O-medium environment, and ^18^O-labeled volatile organic compounds (VOCs) were identified via thermal desorption-gas chromatography/high-resolution mass spectrometry (TD-GC/HRMS), yielding 21 labeled candidates (Fig. 1b, Extended Data Fig. 1a). In Screening-2, HepG2 cells were incubated overnight with FA20:4-d_5_—labeled at the ω-terminal structure. Subsequently, ferroptosis was induced using RAS-selective lethal small molecule 3 (RSL3). Five d_5_-labeled VOCs were detected (Fig. 1c, Extended Data Fig. 1b, Extended Data Fig. 2). Isotope-labeled VOLs were detected exclusively under conditions in which both isotope labeling and RSL3 treatment were applied. Two VOCs, 1-octen-3-ol and 2-pentylfuran, were identified using both screening methods (Fig. 1d). The production of these markers increased in response to ferroptosis inducers (RSL3 and imidazole ketone erastin [IKE]) and decreased with liproxstatin-1 (Lip-1) co-treatment (Fig. 1e).

The chemical pathways responsible for the formation of 1-octen-3-ol and 2-pentylfuran from FA20:4 were also investigated. While 2-pentylfuran is presumed to form via intramolecular cyclization of 4-hydroxy-2-nonenal (a product of iron-dependent LPO of ω-6 PUFAs) [8], the precise mechanism of 1-octen-3-ol generation has not been experimentally established (Fig. 1f and g). Accordingly, *in vitro* reactions were performed using PUFA peroxides and hemin. Among the tested substrates (15-HpETE, 12-HpETE, 9-HpODE, and 13-HpODE), only 12-HpETE, which bears a ω-9 peroxide structure—yielded 1-octen-3-ol (Fig. 1h and i). This was accompanied by the accumulation of key intermediates, including fragmented fatty acids (C_12_H_16_O_3_) and octenyl radicals (•C_8_H_15_) (Fig. 1j).

These findings reveal a previously uncharacterized mechanism for 1-octen-3-ol formation involving sequential Fenton reactions and β-scission from FA20:4-derived 12-HpETE. This establishes a direct mechanistic link between ω-9 lipid peroxidation and the generation of 1-octen-3-ol, a ferroptosis-associated volatile marker.

### 3.2 Ferroptotic VOLs generated via iron-driven oxidative cleavage of polyunsaturated fatty acids

To further elucidate the chemical sources of 1-octen-3-ol and 2-pentylfuran beyond arachidonic acid, a series of *in vitro* oxidation experiments were performed on several PUFAs under iron-dependent conditions.

By systematically analyzing VOLs produced in these reactions with solid-phase microextraction (SPME)-GC/MS (Fig. 2a), a reference library was constructed that mapped each PUFA to its characteristic iron-dependent oxidation products. This defines the broader chemical landscape of iron-dependent VOL formation (Fig. 2b). This approach revealed that 1-octen-3-ol and 2-pentylfuran were consistently produced via the iron-driven cleavage of ω-6 PUFAs (Fig. 2c). Meanwhile, ω-3 PUFAs generated 2-ethylfuran via the same iron-dependent oxidation pathway (Fig. 2d; Extended Data Fig. 3).

These findings were further validated in ferroptosis-induced cells using deuterium-labeled PUFAs (**Extended Data** Fig. 4). After incorporating each labeled PUFA into the cell membranes, ferroptosis was induced, and SPME-GC/MS was performed to detect the resulting d-labeled VOLs (Fig. 2e). The quantitative GC/MS ions for these labeled VOLs were defined based on the *in vitro* oxidation of the corresponding d-PUFAs (**Extended Data** Fig. 5). The incorporation of FA18:2-d_4_ (another ω-6 PUFA) significantly increased the levels of d-labeled 1-octen-3-ol and 2-pentylfuran levels (Fig. 2f). Similarly, the incorporation of DHA-d_5_ (an ω-3 PUFA) led to the production of d-labeled 2-ethylfuran (Fig. 2g).

Overall, 1-octen-3-ol, 2-pentylfuran, and 2-ethylfuran were identified as key ferroptosis-associated VOL markers derived from distinct PUFA classes. This comprehensive library-based strategy established a clear link between specific PUFAs, iron-dependent oxidative pathways, and the generation of signature VOLs during ferroptosis.

### 3.3 Gas-phase accumulation of specific VOLs reflects ferroptosis progression

Next, the ability of the three identified VOLs—1-octen-3-ol, 2-pentylfuran, and 2-ethylfuran—to accumulate in the gas phase alongside ferroptosis progression was evaluated. Cells treated with the ferroptosis inducer RSL3 were analyzed at multiple times to measure lactate dehydrogenase (LDH) release, intracellular oxidized lipid levels, and VOLs. Considering that these compounds arise via iron-dependent LPO, the cells were co-treated with Lip-1 or the iron chelator deferoxamine (DFO). LDH release—a classic marker of cell death—increased significantly 4 h after RSL3 treatment (Fig. 3a). Highly oxidized phospholipids (e.g., PE38:4; O3, **Extended Data** Fig. 6) appeared as early as 2 h (Fig. 3b and c); however, their formation was blocked by Lip-1 or DFO. Notably, the three VOLs appeared in the gas phase within 2 h of RSL3 treatment (Fig. 3d), coinciding with the onset of iron-dependent LPO activity. This effect was suppressed by Lip-1 and DFO (Fig. 3f). Thus, these VOLs reliably reflect the progression of ferroptotic cell death as gas-phase markers.

The three VOLs were elevated by various ferroptosis inducers (FINO2 and ML162) in multiple cell lines (Calu-1 and HEK293; Fig. 3f and g; Extended Data Fig. 7a and b). In contrast, apoptosis triggered by staurosporine or etoposide (Fig. 3h) failed to increase the abundance of these proteins (Fig. 3i). Furthermore, conditions such as etoposide-induced senescence or oleic/linoleic acid–induced lipid accumulation—where mild oxidative stress and LPO can occur without cell death [9,10]—did not elevate the VOLs’ levels (Fig. 3j and k, Extended Data Fig. 8).

These results demonstrate that 1-octen-3-ol, 2-pentylfuran, and 2-ethylfuran accumulate in the gas phase only when iron-dependent LPO drives ferroptosis and remain unchanged under other types of cell death or non-lethal oxidative stress. Hence, these VOLs are potentially robust gas-phase markers for real-time monitoring of ferroptosis.

### 3.4 Hepatic ferroptosis induces 1-octen-3-ol, 2-pentylfuran, and 2-ethylfuran production and release as specific volatile markers

To validate whether the ferroptotic VOL signature previously established in *in vitro* cell experiments is also released as gas from living organisms, we performed *in vivo* animal experiments using a mouse model of liver disease (Fig. 4). The liver is prone to ferroptosis owing to its high iron content, and ferroptosis is implicated in the progression of drug-induced acute hepatitis [11]. Therefore, mouse models of liver disease were employed to determine whether the ferroptotic VOL signature discovered *in vitro* translates into ferroptosis-related VOLs produced in the liver *in vivo* and detected in the emitted animal-derived gas.

First, an acetaminophen (APAP)-induced acute liver failure (ALF) model was used. High-dose APAP depletes glutathione (GSH), leading to extensive hepatocellular ferroptosis [12]. Male C57BL/6j mice were fasted and treated with APAP, and gas sampling and biochemical analyses were performed 4 h later (Fig. 4a). The effect of N-acetylcysteine (NAC)—a GSH precursor—was also evaluated. APAP significantly elevated plasma alanine aminotransferase (ALT) activity and increased oxidized lipids in the liver tissue, indicating liver injury and ferroptosis (Fig. 4b–d). However, NAC administration attenuated these effects. Notably, 1-octen-3-ol, 2-pentylfuran, and 2-ethylfuran levels increased in APAP-treated liver tissue and decreased upon NAC administration (Fig. 4e).

Subsequently, the mice were housed in sealed cages perfused with a VOC-free gas mixture (N_2_:78 %, O_2_:22 %, 50 mL/min/cage), and the effluent gas was collected for 1 h using bio-monitoring inert-coated tubes for TD-GC/MS analysis (Fig. 4f). Elevated levels of 1-octen-3-ol, 2-pentylfuran, and 2-ethylfuran were detected in the gas released from APAP-treated mice, which decreased after NAC treatment (Fig. 4g). Thus, ferroptosis in the APAP-ALF model can be monitored noninvasively via VOL markers in the gas derived from mice.

Ferroptosis also contributes to metabolic dysfunction-associated steatohepatitis (MASH) [13]. Hence, the production of the three ferroptosis VOLs was assessed in a MASH mouse model that fed a choline-deficient, l-amino acid-defined, high-fat diet (CDAHFD) for 6 weeks (Fig. 4h). Vitamin E (VE), a known ferroptosis inhibitor, was administered to a subset of mice. CDAHFD-fed mice showed marked hepatic fat accumulation, elevated plasma ALT levels, and increased oxidized phospholipids, indicative of progressive liver injury and ferroptosis. VE supplementation ameliorated these effects. Additionally, the ferroptosis VOL signature was significantly higher in the liver tissue of the CDAHFD group and lower in the VE-supplemented group (Fig. 4i–k, and Extended Data Fig. 9). The levels of these markers remained low in non-liver tissues, underscoring their specific origin in ferroptotic hepatocytes (Fig. 4l–n). Furthermore, gas analysis confirmed elevated levels of 1-octen-3-ol and 2-pentylfuran in CDAHFD-fed mice and their reduction following VE intervention. This suggests that ferroptosis in the liver can be detected based on the levels of these VOL markers (Fig. 4o).

Collectively, these findings confirm that the ferroptosis-associated VOLs identified *in vitro*—1-octen-3-ol, 2-pentylfuran, and 2-ethylfuran—are also emitted *in vivo* via hepatic ferroptosis. Thus, these VOL markers enable the noninvasive detection of ferroptosis-driven liver injury in acute and chronic disease models.

### 3.5 Noninvasive detection of ferroptosis in patients with MASLD using breath analysis

Finally, we investigated whether the identified VOLs were elevated in the exhaled breath of patients with MASLD, including metabolic dysfunction-associated steatohepatitis (MASH). Breath VOL analysis was conducted on healthy individuals (*n* = 48), patients with MASLD (*n* = 56, comprising MASH without cirrhosis [LC]), and MASLD-related LC patients (*n* = 11) following the collection of breath samples in thermal desorption tubes (Fig. 5a) and TD-GC/MS analysis (Fig. 5b). The ferroptosis VOL markers showed marked variations among the disease groups (Fig. 5c). Patients with MASLD had significantly higher ferroptosis-related VOLs levels (Fig. 5d). Specifically, 1-octen-3-ol levels were elevated in patients with MASLD compared to those in healthy individuals (*p* = 0.0143); however, this increase was suppressed in patients with LC. Meanwhile, 2-pentylfuran levels were elevated in MASLD and LC (*p* = 0.0022 for healthy vs. MASLD, *p* < 0.0001 for healthy vs. LC, and *p* < 0.0001 for MASLD vs. LC). In addition, 2-ethylfuran was significantly elevated in the LC group (*p* < 0.0001 for healthy vs. LC and *p* = 0.0008 for MASLD vs. LC). Combining these markers enabled discrimination between healthy individuals and those with MASLD or LC with area under the curve (AUC) values of 0.824 and 0.931, respectively (Fig. 5e). Furthermore, integrating ferroptosis VOL markers with standard clinical markers (using least absolute shrinkage and selection operator [LASSO] analysis) distinguished MASLD from LC with high confidence (AUC >0.9; Fig. 5f and Extended Data Fig. 10).

Correlations between these VOLs and pathophysiological indices were also evaluated, revealing that 2-pentylfuran showed a moderate correlation (r > 0.4) with fibrosis markers (FIB-4, MAC-2 binding protein glycosylation isomer [M2BPGi]) and the albumin-bilirubin (ALBI) score (r > 0.6) (Fig. 5g). Finally, to assess whether human breath VOLs reflect hepatic oxidized lipid levels, oxidized phosphatidylcholine (oxPC), and oxidized triglyceride (oxTG), which were measured in liver biopsy samples, revealed a strong correlation with breath 2-pentylfuran levels (Fig. 5h).

Collectively, these findings indicate that noninvasive breath analysis of ferroptosis VOLs can identify advanced MASLD stages. Hence, these ferroptosis-derived VOL markers could serve as real-time indicators of disease progression and offer a noninvasive screening strategy for patients with MASLD and LC.

## 4 Discussion

**Fig. 6:**
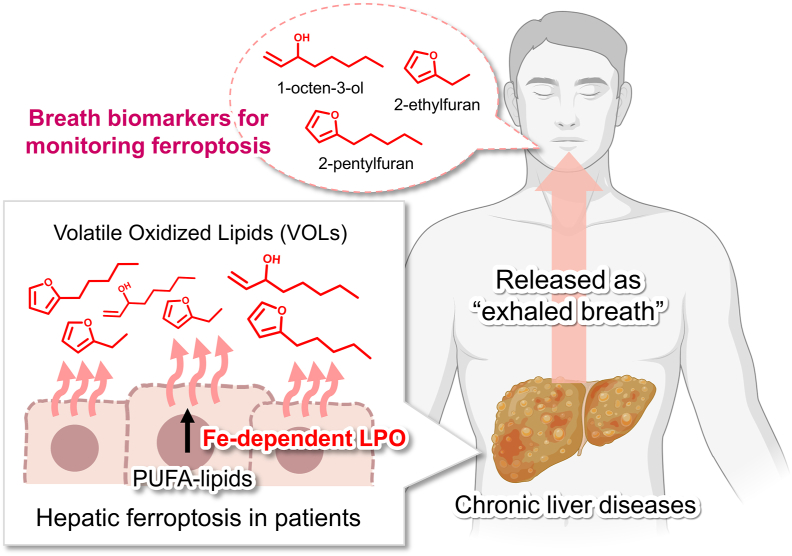
**Schematic illustration of the present study.** VOL markers—1-octen-3-ol, 2-pentylfuran, and 2-ethylfuran—generated via iron-dependent lipid peroxidation and detectable in exhaled breath could serve as real-time, non-invasive indicators of *in vivo* ferroptosis and the progression of chronic liver diseases. The figure was created with BioRender (biorender.com).

In this study, we identified small fragments of lipid peroxides released as gas during ferroptosis and defined their molecular characteristics. PUFA-derived VOLs, namely 1-octen-3-ol, 2-pentylfuran, and 2-ethylfuran, were identified as markers of *in vivo* ferroptosis induced via iron-dependent LPO, which may have clinical relevance (Fig. 6).

VOCs are ubiquitous in ambient air, which complicates the accurate detection and identification of cellular-derived VOCs at trace levels owing to background contamination. To address this, we employed stable isotope-labeled molecules, enabling the differentiation of cellular-derived volatile metabolites from environmental contaminants and achieving highly precise analytical results. However, VOCs other than the identified VOL markers, such as ketones and alcohols, may reflect various intracellular metabolic pathways or cellular metabolic conditions. Thus, further elucidation of the biosynthetic pathways and underlying mechanisms responsible for generating these VOCs will significantly advance our understanding of cellular metabolism and improve the specificity of the detection methods.

Standard lipids were oxidized *in vitro*, and 48 VOLs with different chemical structures were detected (Fig. 2 and Supplementary Table 1). The types and amounts of identified VOLs differed significantly with hemin addition (Fig. 2b). Nonenal and hexenal-like alkenals were more abundant when only AAPH was added (**Extended Data** Fig. 11), presumably as the hock cleavage products of lipid peroxides [14]. For example, hock cleavage initiated by the protonation of 11-HpETE produces 3-nonenal and its isomerized form, 2-nonenal (**Extended Data** Fig. 12). In contrast, samples treated with both AAPH and hemin showed increased production of vinyl alcohol and 2-alkylfuran (Fig. 2c and d). 2-alkylfuran is a cyclized molecule derived from 4-hydroxyalkenal. The formation of 4-hydroxyalkenal is facilitated by the Fenton reaction of 4-hydroperoxyalkenal and PUFA hydroperoxides (Fig. 1g). The addition of hemin, which facilitates this reaction, may have increased the amount of 4-hydroxyalkenal produced.

The VOLs 1-octen-3-ol, 2-pentylfuran, and 2-ethylfuran were produced by the iron-dependent oxidation of PUFA, and confirmed as markers of ferroptosis induction *in vitro*, *in vivo*, and human breath. Specifically, 1-octen-3-ol is biosynthesized from FA18:2 by certain fungi and plants using 10-lipoxygenase and hydroperoxide lyase [15], respectively. However, whether homologous enzymes or enzymatic activities exist in mammals and whether a nonenzymatic formation reaction occurs remains unknown. Herein, we identified a reaction scheme in which iron plays a significant role in the nonenzymatic formation of 1-octen-3-ol. Furthermore, these VOL markers were derived from membrane lipids in HepG2 cells during ferroptotic progression (Fig. 2f and g). Notably, 1-octen-3-ol was produced in greater amounts from FA20:4 than from 2-pentylfuran (Fig. 2f). The generation of 1-octen-3-ol preferentially requires a ω-9 hydroperoxide structure, such as 12-HpETE (Fig. 1f). In contrast, FA18:2 is less prone to forming 10-HpODE with a hydroperoxide moiety at the C9 position via radical-mediated pathways [16]. In contrast, the formation of 2-pentylfuran is dependent on the presence of a ω-10 hydroperoxide structure, which can be generated through radical-mediated oxidation of both FA20:4 and FA18:2, corresponding to 11-HpETE and 9-HpODE, respectively. This mechanistic difference likely accounts for the distinct production profiles of 1-octen-3-ol and 2-pentylfuran from each PUFA substrate.

During ferroptosis, labile iron stored in lysosomes is released into the cytosol, resulting in lethal iron-dependent LPO [17]. In addition, the liver is a major organ with iron stores [18], and an increase in free iron levels in the liver tissue may occur during APAP-ALF and MASH induction [19,20]. As these VOLs are selectively produced in ferroptotic cells, particularly hepatocytes, they may be effective markers for differentiating between healthy and diseased states of the liver.

The levels of ferroptosis VOL markers in the breath of patients with MASLD or LC were higher than those in healthy individuals. Previous fundamental research using mouse models has indicated that hepatocyte ferroptosis triggers increased hepatic inflammation in the early stages of MASH development [13]. This study confirmed that ferroptosis progresses during the early stages of chronic hepatitis in human. In contrast, the levels of 2-pentylfuran and 2-ethylfuran were higher in patients with LC, demonstrating their utility in distinguishing LC from other conditions. This could be attributed to their high lipophilicity and accumulation in liver tissue due to the reduced degradative capacity associated with impaired liver function in patients with LC. The differences in the properties of these markers could be attributed to their retention times in the body; for example, lipid accumulation in lipid droplets may increase retention time. Furthermore, 2-pentylfuran is degraded by P450 [21], and as P450 activity is compromised in patients with LC [22], the accumulation of 2-pentylfuran and 2-ethylfuran produced by ferroptosis in liver tissue may be more pronounced in these patients. Although further detailed investigation of the pharmacokinetics of VOLs is necessary, these results suggest that combining the three VOL markers could be useful for diagnosing MASLD/LC stages and identifying patients (**Extended Data** Fig. 13).

MASLD, with an estimated global prevalence of 30 %, is the most rapidly growing contributor to liver-related morbidity and mortality [23,24]. Due to its prevalence and close association with the global epidemics of obesity and metabolic syndrome, an easily obtained, noninvasive, and liver-specific biomarker capable of identifying individuals with high-risk MASLD or MASH is among the most challenging unmet clinical needs [25]. In this study, we demonstrated that ferroptosis-related VOLs may be useful as liver-specific mechanistic biomarkers for MASLD. Although hepatic histopathological improvements are necessary for drug development for MASH, the invasive nature of percutaneous liver biopsies, along with their high inter- and intra-investigator variability, are considerable obstacles [26]. Although further investigations are warranted, our technology for analyzing ferroptosis-related VOLs in exhaled breath may prove effective for mass screening, risk stratification, and assessing therapeutic efficacy.

However, the bioactivities of these VOLs remain unknown and require further investigation. Intracellular molecules released from ferroptotic cells induce ferroptosis in surrounding cells [27]. Low-molecular-weight oxidized lipids, including VOLs, are implicated in ferroptosis propagation. Interestingly, 1-octen-3-one (the oxidized form of 1-octen-3-ol) was highly cytotoxic (**Extended Data** Fig. 14). Similarly, α,β-unsaturated carbonyls, including 1-octen-3-one, are highly electrophilic and cause modifications of nucleophilic amino acid residues, leading to ferroptosis [7]. In addition, compounds containing a furan ring structure, such as 2-pentylfuran and 2-ethylfuran, have also been reported to contribute to the induction of ferroptosis [28]. In addition, their precursor, 4-hydroxynonenal (4-HNE), promotes ferroptosis by downregulating the expression of GPx4 [29,30]. Overall, the VOLs produced during ferroptosis and diffused into the environment may contribute to ferroptosis in neighboring cells. Therefore, the bioactivity of the VOLs identified in this study, which are selective for ferroptotic cells, warrants further investigation.

A major limitation of using VOL markers in exhaled breath is the unclear origin of VOLs. In this study, we confirmed that the three marker VOLs were specifically produced in the liver of a mouse APAP-ALF/MASH model. However, the organ specificity of VOL sources in human MASLD/LC requires further investigation. In addition, changes in VOL production patterns in the liver due to comorbidities should be characterized. Lipid peroxidation is enhanced in patients with obesity and diabetes [31,32], and alterations in the levels of certain exhaled VOC have also been observed in these populations [33,34]. Additionally, lifestyle factors such as smoking have been reported to influence the profile of exhaled VOCs [35]. Therefore, changes in VOL production associated with lifestyle-related comorbidities such as obesity and diabetes, as well as behavioral and extrahepatic factors such as smoking, alcohol consumption, and pulmonary diseases, must be assessed in large-scale studies. In this study, we analyzed the correlation between the identified VOL markers and smoking habits and the presence of pulmonary diseases; however, no significant associations were observed with either factor (**Extended Data** Fig. 15). In addition, further investigation is warranted to evaluate the *in vivo* specificity of this marker for ferroptosis using models in which ferroptosis is the exclusive mode of cell death, such as Gpx4-knockout mice.

The potential influence of dietary lipids and pharmacological interventions on exhaled VOLs warrants further investigation. Since VOL profiles vary depending on the type of precursor lipids (e.g., ω-3 PUFAs vs. ω-6 PUFAs) (Fig. 2), the specific VOLs to be targeted may differ according to the subject's dietary habits, which in turn shape their internal lipid profile. Therefore, to establish a more robust detection system, it is important to identify combinations of VOL markers or correction strategies that minimize these confounding effects. In addition to nonenzymatic reactions, lipid fragmentation products can be generated enzymatically via lipid-oxidizing enzymes, such as lipoxygenases (LOX) and cyclooxygenases (COX) [36,37]. Accordingly, the impact of medications targeting these enzymes, such as non-steroidal anti-inflammatory drugs (NSAIDs), on the VOL profile should be carefully examined.

Detection technologies and clinical diagnostic methods targeting exhaled breath allow noninvasive examination of the physiological status of an individual. However, their development has lagged significantly behind that of liquid biopsy technologies that utilize blood and other bodily fluids. The rate-limiting step in their development is the identification of gas marker molecules that are specific to biological phenomena or diseases. Therefore, using this study as a model, gas markers can be explored as new indicators of metabolic reactions. This can facilitate the development of detection technologies and clinical diagnostics.

## 5 Conclusions

We employed an innovative analytical platform, oxidative volatolomics, to identify VOLs (1-octen-3-ol, 2-ethylfuran, and 2-pentylfuran) specifically released during ferroptosis. Using these markers, we developed a technology for the noninvasive detection of ferroptosis *in vivo*. We anticipate that the markers and detection technologies developed in this study will be utilized and further developed, contributing to the noninvasive and early detection of ferroptosis-related diseases.

## CRediT authorship contribution statement

**Yuta Matsuoka:** Writing – review & editing, Writing – original draft, Validation, Project administration, Methodology, Investigation, Funding acquisition, Formal analysis, Data curation, Conceptualization. **Yoshinori Katsumata:** Writing – original draft, Validation, Resources, Investigation. **Po-sung Chu:** Validation, Resources, Investigation. **Rei Morikawa:** Validation, Resources, Investigation. **Nobuhiro Nakamoto:** Validation, Resources, Investigation. **Kohta Iguchi:** Validation, Resources, Investigation. **Ken Takahashi:** Validation, Resources, Investigation. **Tadayuki Kou:** Validation, Resources, Investigation. **Ryo Ito:** Validation, Resources, Investigation. **Kojiro Taura:** Validation, Investigation, Conceptualization. **Shujiro Yazumi:** Validation, Resources, Investigation. **Hiroaki Terajima:** Validation, Resources, Investigation. **Gen Honjo:** Validation, Resources, Investigation. **Genki Ichihara:** Validation, Resources, Investigation. **Yuki Muramoto:** Validation, Resources, Investigation. **Kazuki Sato:** Validation, Resources, Investigation. **Rae Maeda:** Validation, Investigation. **Kazuhiro Hata:** Validation. **Naoya Toriu:** Validation. **Motoko Yanagita:** Validation. **Masaki Tajima:** Investigation. **Sidonia Fagarasan:** Writing – review & editing. **Ken-ichi Yamada:** Writing – review & editing. **Yuki Sugiura:** Writing – review & editing, Writing – original draft, Supervision.

## Data availability statement

Data will be made available on request. The raw and processed MS data generated in this study are publicly available in the Metabolomics Workbench repository under accession doi: 10.21228/M8JK13.

## Declaration of competing interest

The authors declare that they have no known competing financial interests or personal relationships that could have appeared to influence the work reported in this paper.
