# Supplementary material for: Monitoring ferroptosis in vivo: Iron-driven volatile oxidized lipids as breath biomarkers
Source: Redox Biol. 2025 Sep 2;86:103858. doi: 10.1016/j.redox.2025.103858 (PMC12454666; doi:10.1016/j.redox.2025.103858)
Supplement: Multimedia component 1 [file mmc1.pptx]

## Slide 1
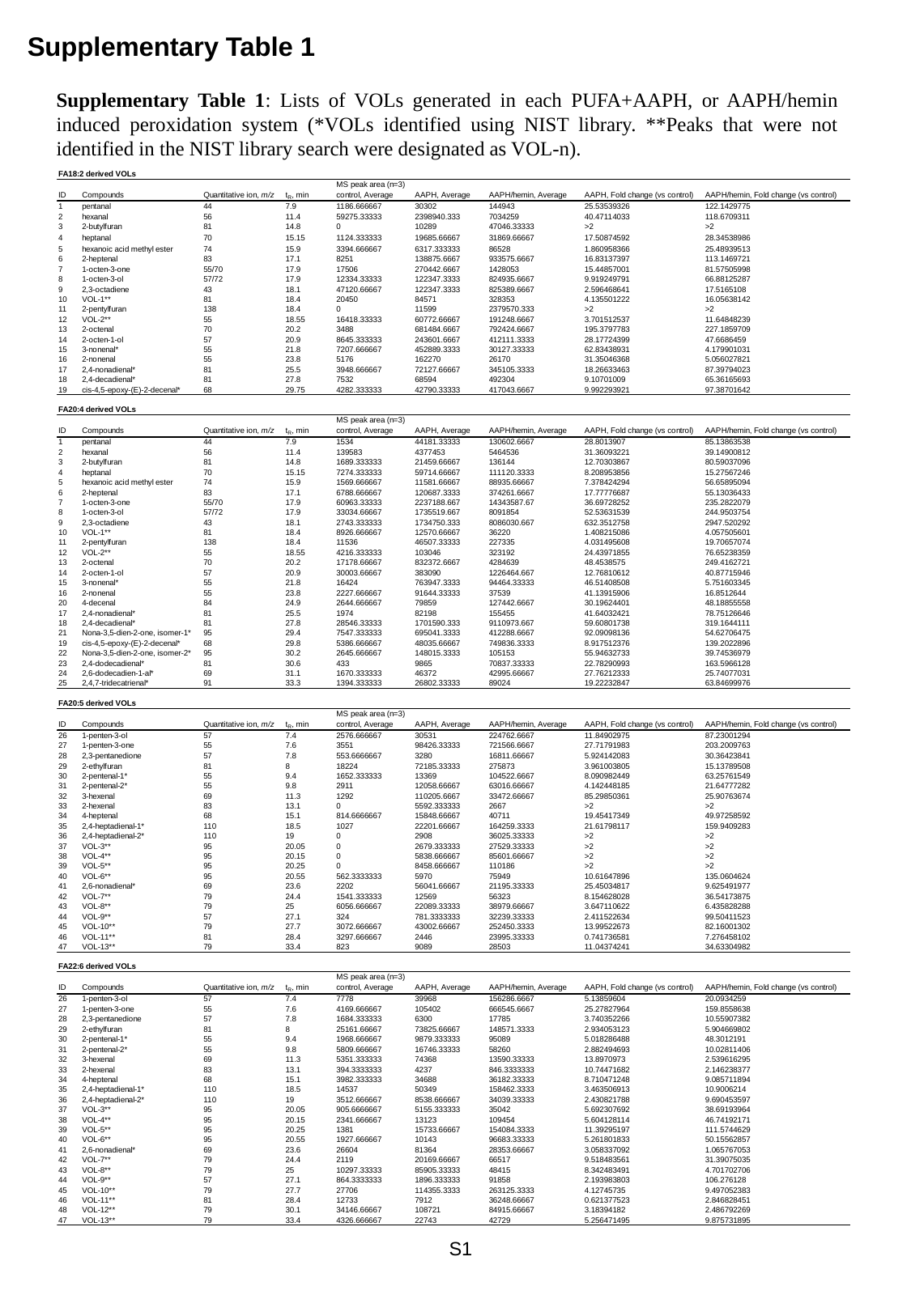

Supplementary Table 1
Supplementary Table 1: Lists of VOLs generated in each PUFA+AAPH, or AAPH/hemin induced peroxidation system (*VOLs identified using NIST library. **Peaks that were not identified in the NIST library search were designated as VOL-n).
S1

## Slide 2
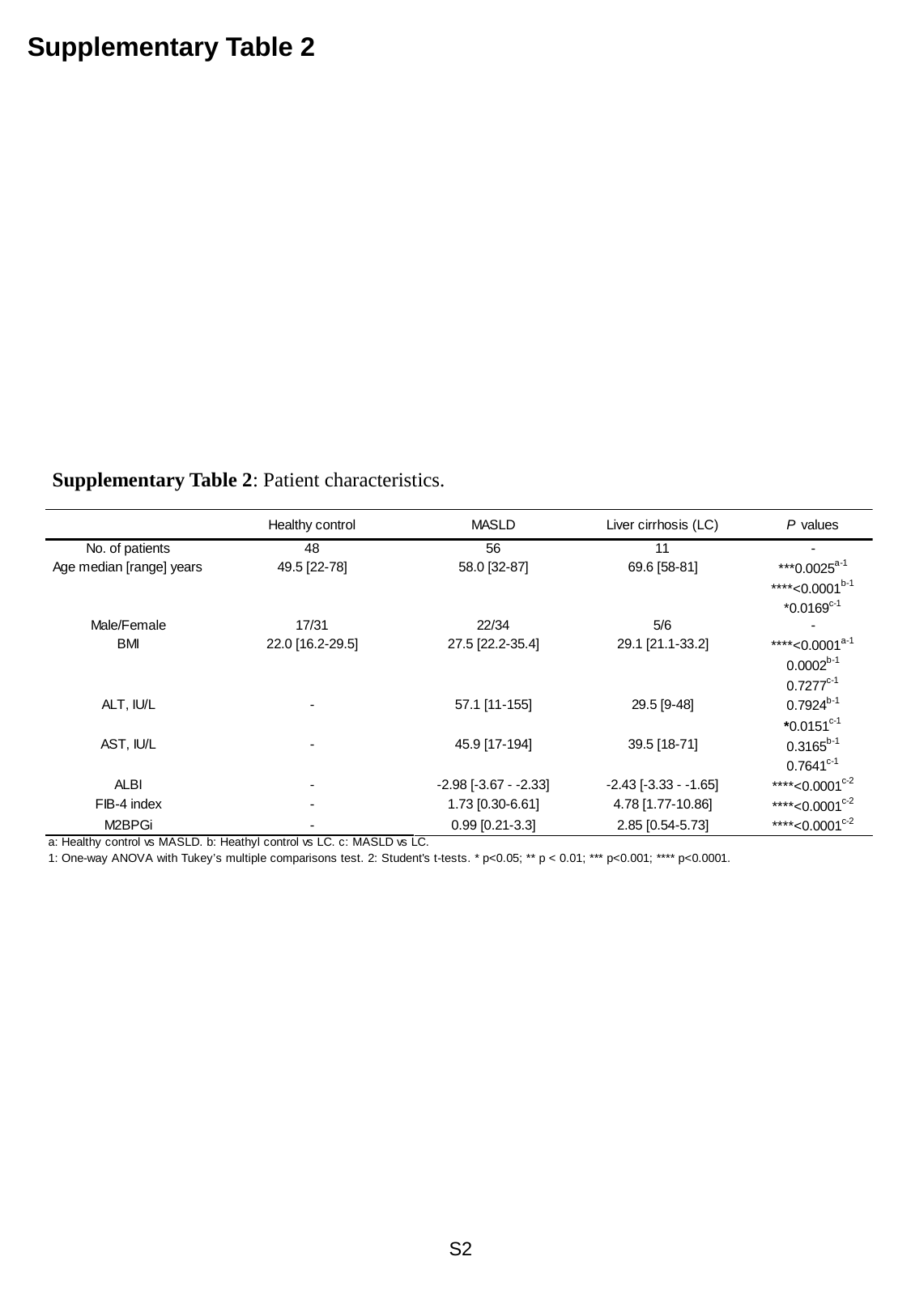

Supplementary Table 2
Supplementary Table 2: Patient characteristics.
S2

## Slide 3
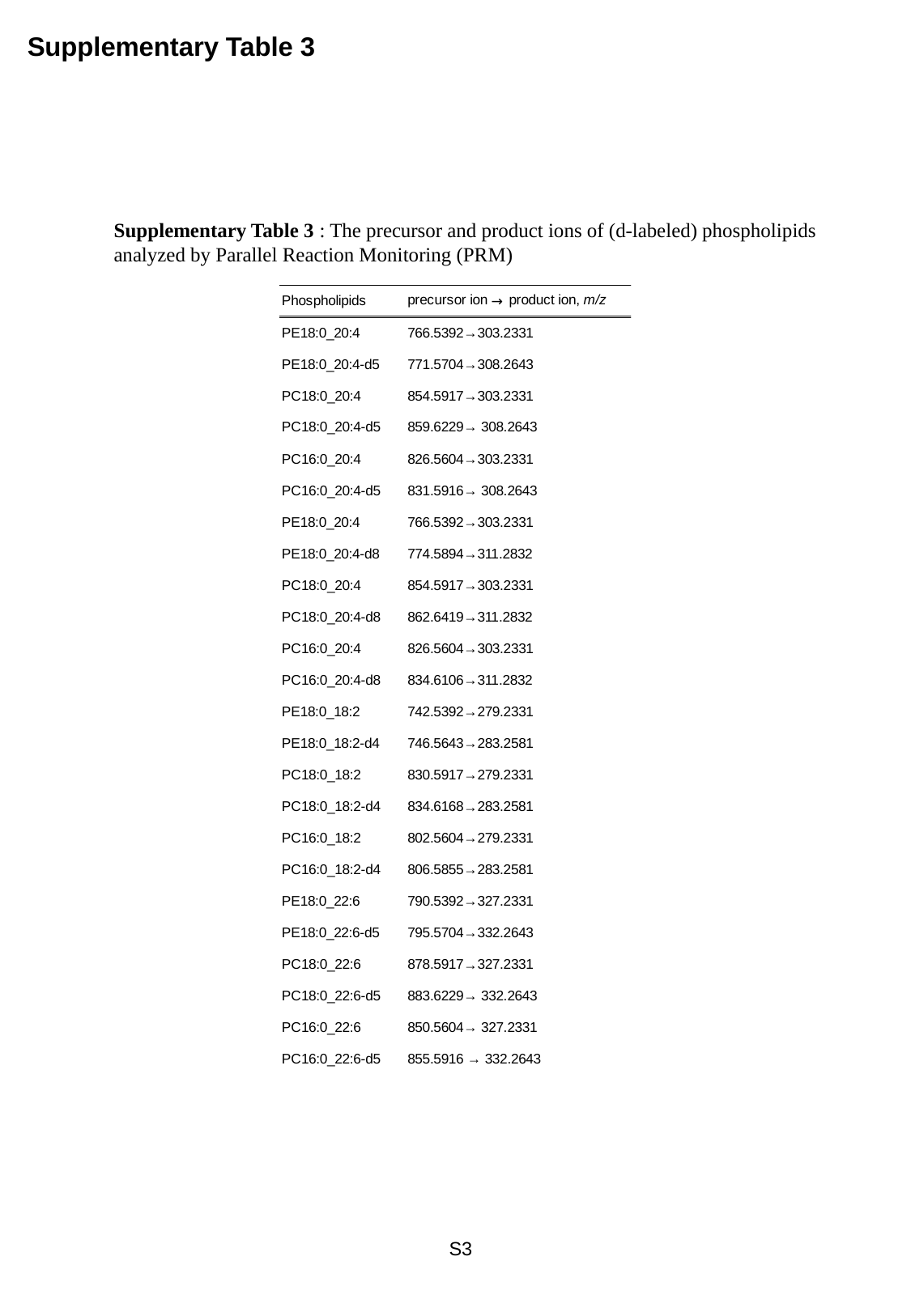

Supplementary Table 3
Supplementary Table 3 : The precursor and product ions of (d-labeled) phospholipids analyzed by Parallel Reaction Monitoring (PRM)
S3

## Slide 4
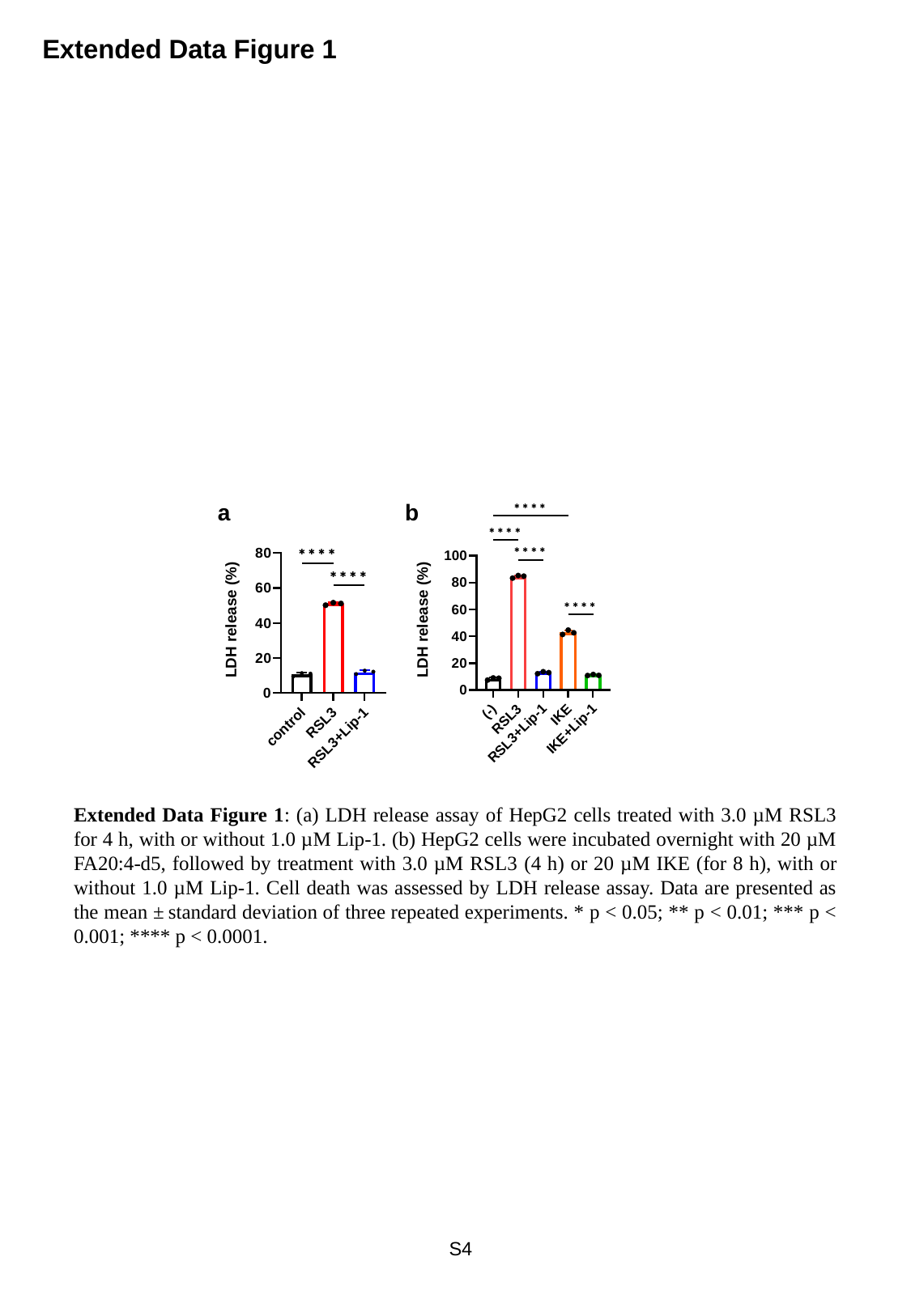

Extended Data Figure 1
a
b
LDH release (%)
LDH release (%)
Extended Data Figure 1: (a) LDH release assay of HepG2 cells treated with 3.0 µM RSL3 for 4 h, with or without 1.0 µM Lip-1. (b) HepG2 cells were incubated overnight with 20 µM FA20:4-d5, followed by treatment with 3.0 µM RSL3 (4 h) or 20 µM IKE (for 8 h), with or without 1.0 µM Lip-1. Cell death was assessed by LDH release assay. Data are presented as the mean ± standard deviation of three repeated experiments. * p < 0.05; ** p < 0.01; *** p < 0.001; **** p < 0.0001.
S4

## Slide 5
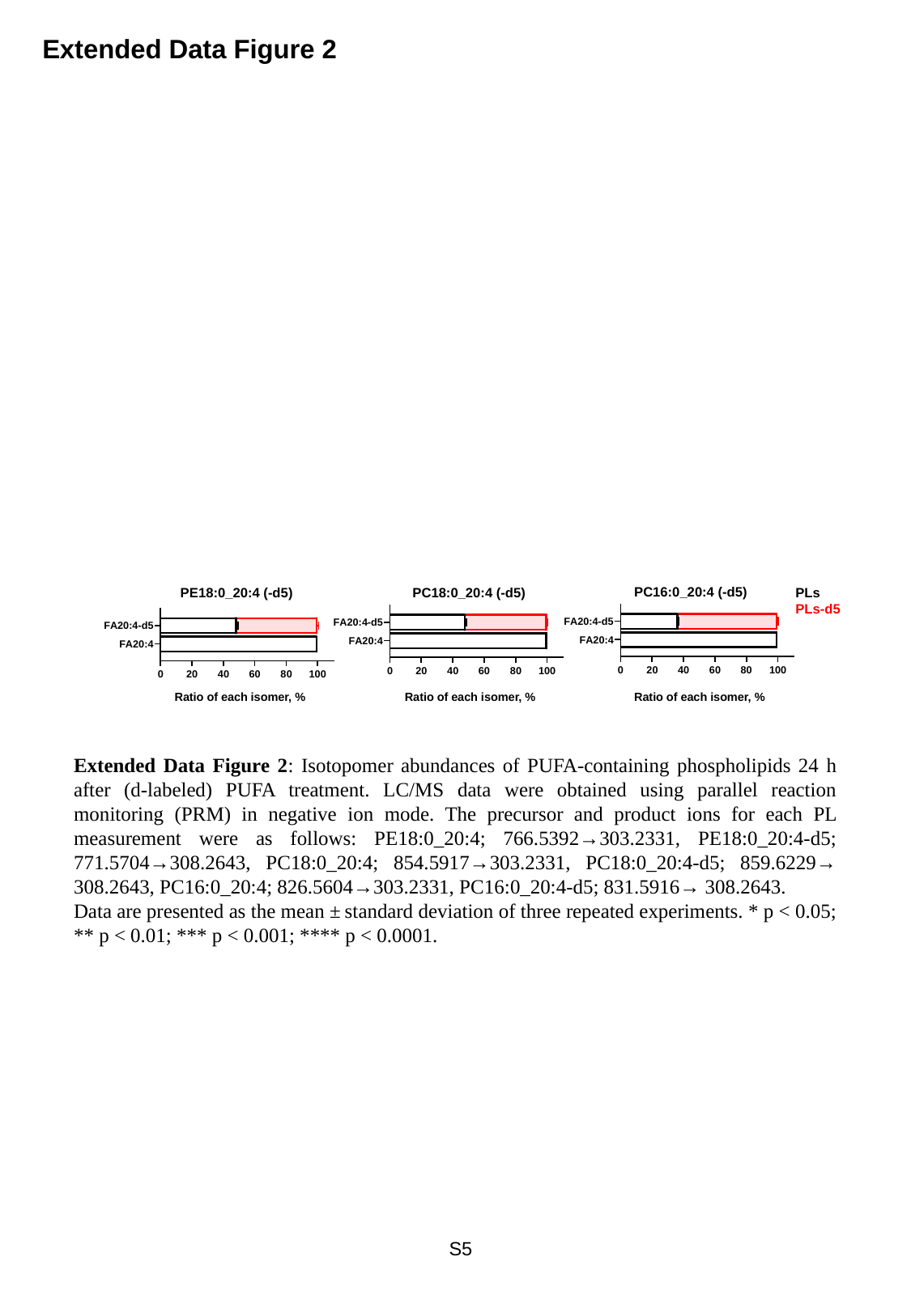

Extended Data Figure 2
PC16:0_20:4 (-d5)
PLs
PLs-d5
PE18:0_20:4 (-d5)
PC18:0_20:4 (-d5)
Ratio of each isomer, %
Ratio of each isomer, %
Ratio of each isomer, %
Extended Data Figure 2: Isotopomer abundances of PUFA-containing phospholipids 24 h after (d-labeled) PUFA treatment. LC/MS data were obtained using parallel reaction monitoring (PRM) in negative ion mode. The precursor and product ions for each PL measurement were as follows: PE18:0_20:4; 766.5392→303.2331, PE18:0_20:4-d5; 771.5704→308.2643, PC18:0_20:4; 854.5917→303.2331, PC18:0_20:4-d5; 859.6229→ 308.2643, PC16:0_20:4; 826.5604→303.2331, PC16:0_20:4-d5; 831.5916→ 308.2643.
Data are presented as the mean ± standard deviation of three repeated experiments. * p < 0.05; ** p < 0.01; *** p < 0.001; **** p < 0.0001.
S5

## Slide 6
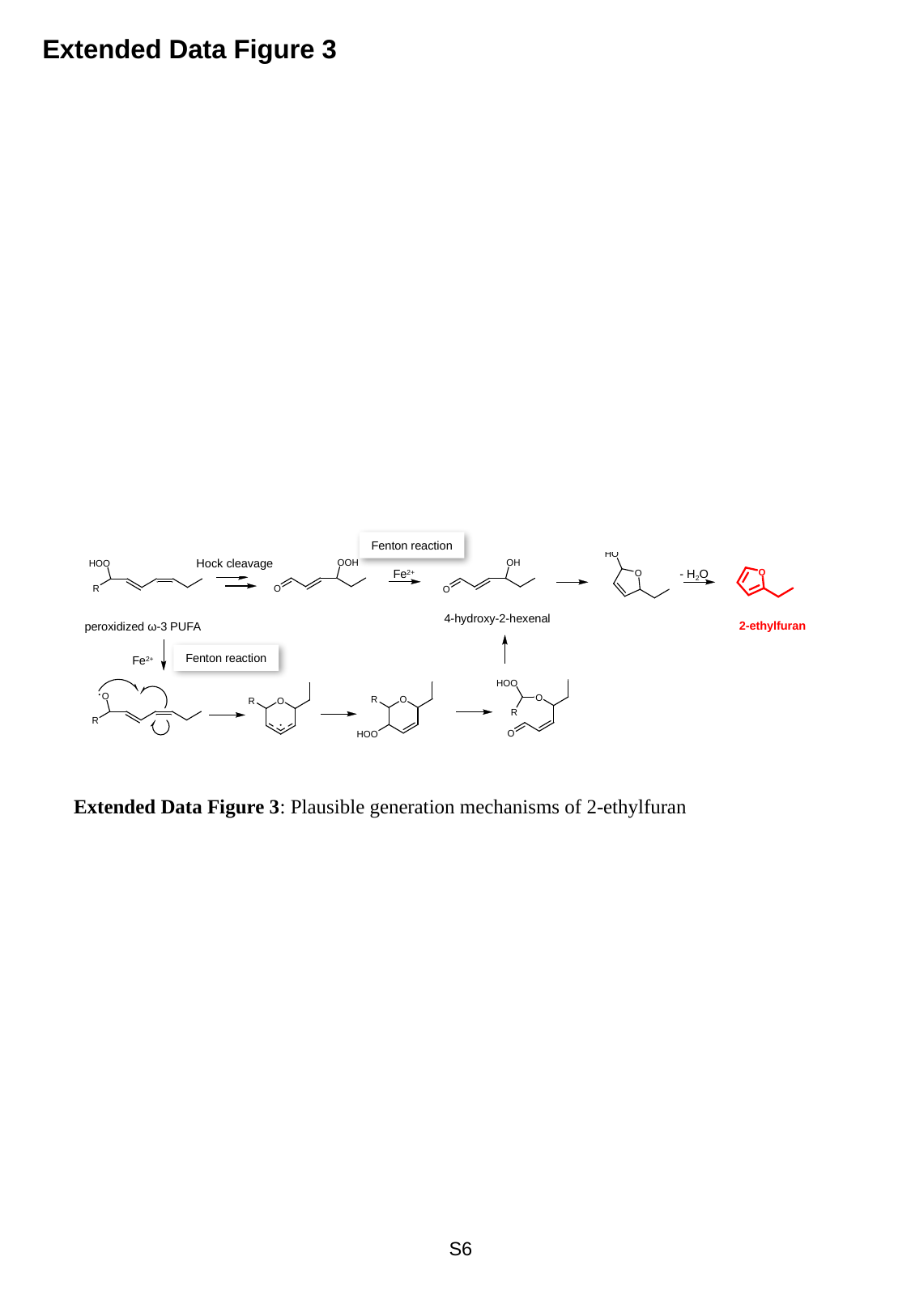

Extended Data Figure 3
Fenton reaction
Hock cleavage
- H2O
Fe2+
4-hydroxy-2-hexenal
2-ethylfuran
peroxidized ω-3 PUFA
Fenton reaction
Fe2+
Extended Data Figure 3: Plausible generation mechanisms of 2-ethylfuran
S6

## Slide 7
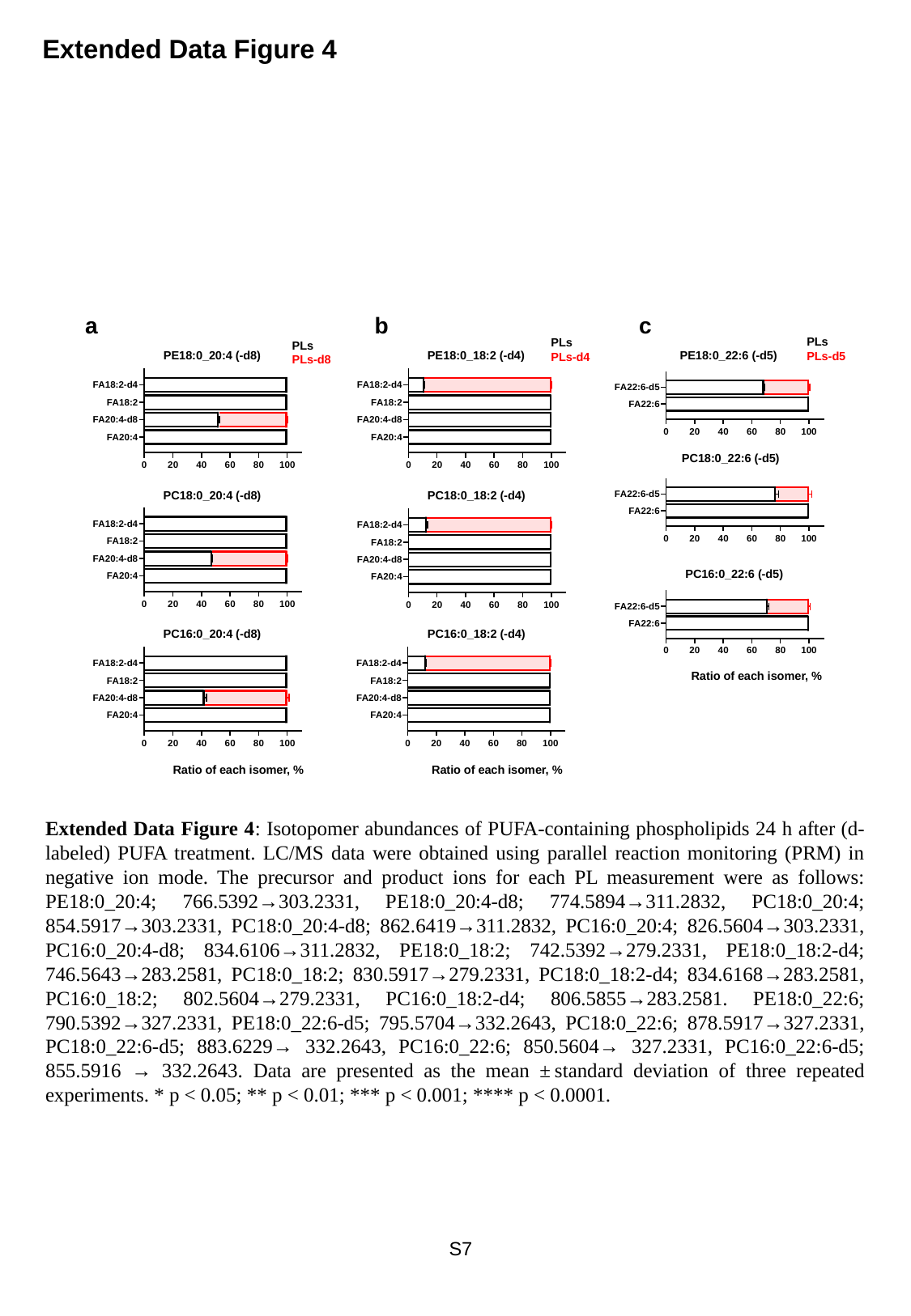

Extended Data Figure 4
a
b
c
PLs
PLs-d5
PLs
PLs-d4
PLs
PLs-d8
PE18:0_22:6 (-d5)
PE18:0_20:4 (-d8)
PE18:0_18:2 (-d4)
PC18:0_22:6 (-d5)
PC18:0_20:4 (-d8)
PC18:0_18:2 (-d4)
PC16:0_22:6 (-d5)
PC16:0_18:2 (-d4)
PC16:0_20:4 (-d8)
Ratio of each isomer, %
Ratio of each isomer, %
Ratio of each isomer, %
Extended Data Figure 4: Isotopomer abundances of PUFA-containing phospholipids 24 h after (d-labeled) PUFA treatment. LC/MS data were obtained using parallel reaction monitoring (PRM) in negative ion mode. The precursor and product ions for each PL measurement were as follows: PE18:0_20:4; 766.5392→303.2331, PE18:0_20:4-d8; 774.5894→311.2832, PC18:0_20:4; 854.5917→303.2331, PC18:0_20:4-d8; 862.6419→311.2832, PC16:0_20:4; 826.5604→303.2331, PC16:0_20:4-d8; 834.6106→311.2832, PE18:0_18:2; 742.5392→279.2331, PE18:0_18:2-d4; 746.5643→283.2581, PC18:0_18:2; 830.5917→279.2331, PC18:0_18:2-d4; 834.6168→283.2581, PC16:0_18:2; 802.5604→279.2331, PC16:0_18:2-d4; 806.5855→283.2581. PE18:0_22:6; 790.5392→327.2331, PE18:0_22:6-d5; 795.5704→332.2643, PC18:0_22:6; 878.5917→327.2331, PC18:0_22:6-d5; 883.6229→ 332.2643, PC16:0_22:6; 850.5604→ 327.2331, PC16:0_22:6-d5; 855.5916 → 332.2643. Data are presented as the mean ± standard deviation of three repeated experiments. * p < 0.05; ** p < 0.01; *** p < 0.001; **** p < 0.0001.
S7

## Slide 8
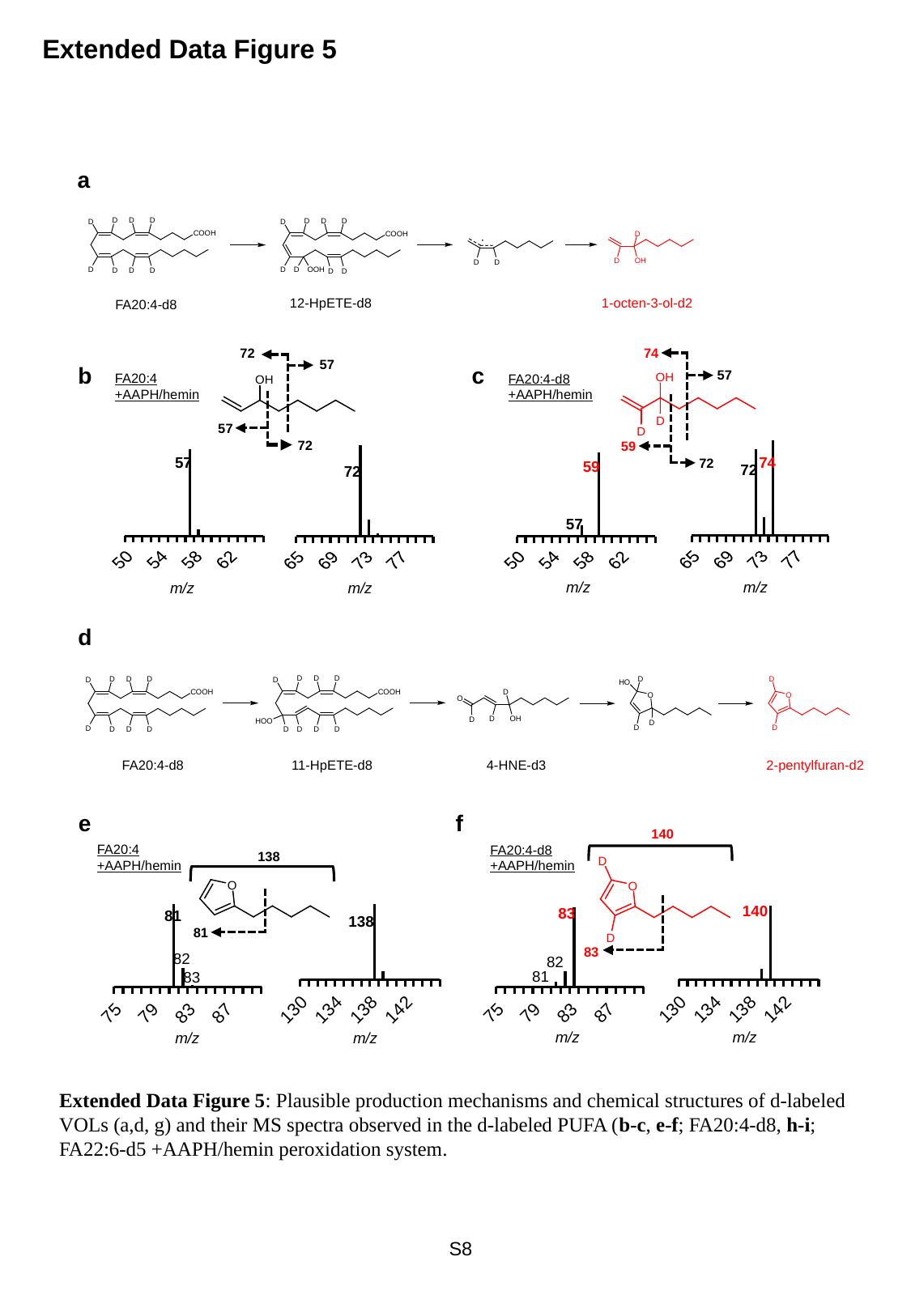

Extended Data Figure 5
a
1-octen-3-ol-d2
12-HpETE-d8
FA20:4-d8
72
74
57
b
c
57
FA20:4
+AAPH/hemin
FA20:4-d8
+AAPH/hemin
57
### Chart
| Category | |
|---|---|
| 65 | 0.0 |
| 66 | 0.0 |
| 67 | 0.0 |
| 68 | 0.0 |
| 69 | 0.0 |
| 70 | 0.0 |
| 71 | 0.0 |
| 72 | 3016.4228515625 |
| 73 | 652.398193359375 |
| 74 | 3327.2080078125 |
| 75 | 0.0 |
| 76 | 0.0 |
| 77 | 0.0 |
| 78 | 0.0 |
| 79 | 0.0 |
| 80 | 0.0 |
### Chart
| Category | |
|---|---|
| 65 | 0.0 |
| 66 | 0.0 |
| 67 | 0.0 |
| 68 | 0.0 |
| 69 | 0.0 |
| 70 | 0.0 |
| 71 | 0.0 |
| 72 | 5456.681640625 |
| 73 | 984.672180175781 |
| 74 | 189.402236938477 |
| 75 | 0.0 |
| 76 | 0.0 |
| 77 | 0.0 |
| 78 | 0.0 |
| 79 | 0.0 |
| 80 | 0.0 |72
59
### Chart
| Category | |
|---|---|
| 50 | 0.0 |
| 51 | 0.0 |
| 52 | 0.0 |
| 53 | 0.0 |
| 54 | 0.0 |
| 55 | 0.0 |
| 56 | 0.0 |
| 57 | 31778.61328125 |
| 58 | 2440.45043945313 |
| 59 | 321.406616210938 |
| 60 | 0.0 |
| 61 | 0.0 |
| 62 | 0.0 |
| 63 | 0.0 |
| 64 | 0.0 |
| 65 | 0.0 |
### Chart
| Category | |
|---|---|
| 50 | 0.0 |
| 51 | 0.0 |
| 52 | 0.0 |
| 53 | 0.0 |
| 54 | 0.0 |
| 55 | 0.0 |
| 56 | 0.0 |
| 57 | 4016.91845703125 |
| 58 | 0.0 |
| 59 | 30553.240234375 |
| 60 | 0.0 |
| 61 | 0.0 |
| 62 | 0.0 |
| 63 | 0.0 |
| 64 | 0.0 |
| 65 | 0.0 |74
57
72
59
72
72
57
m/z
m/z
m/z
m/z
d
11-HpETE-d8
4-HNE-d3
FA20:4-d8
2-pentylfuran-d2
e
f
140
FA20:4
+AAPH/hemin
FA20:4-d8
+AAPH/hemin
138
### Chart
| Category | |
|---|---|
| 75 | 0.0 |
| 76 | 0.0 |
| 77 | 0.0 |
| 78 | 0.0 |
| 79 | 0.0 |
| 80 | 0.0 |
| 81 | 39705.1328125 |
| 82 | 9140.365234375 |
| 83 | 711.268859863281 |
| 84 | 0.0 |
| 85 | 0.0 |
| 86 | 0.0 |
| 87 | 0.0 |
| 88 | 0.0 |
| 89 | 0.0 |
| 90 | 0.0 |
### Chart
| Category | |
|---|---|
| 130 | 0.0 |
| 131 | 0.0 |
| 132 | 0.0 |
| 133 | 0.0 |
| 134 | 0.0 |
| 135 | 0.0 |
| 136 | 0.0 |
| 137 | 0.0 |
| 138 | 91.7653656005859 |
| 139 | 783.804260253906 |
| 140 | 5425.296875 |
| 141 | 0.0 |
| 142 | 0.0 |
| 143 | 0.0 |
| 144 | 0.0 |
| 145 | 0.0 |
### Chart
| Category | |
|---|---|
| 75 | 0.0 |
| 76 | 0.0 |
| 77 | 0.0 |
| 78 | 0.0 |
| 79 | 0.0 |
| 80 | 0.0 |
| 81 | 2694.5625 |
| 82 | 8572.8349609375 |
| 83 | 44669.5078125 |
| 84 | 0.0 |
| 85 | 0.0 |
| 86 | 0.0 |
| 87 | 0.0 |
| 88 | 0.0 |
| 89 | 0.0 |
| 90 | 0.0 |140
### Chart
| Category | |
|---|---|
| 130 | 0.0 |
| 131 | 0.0 |
| 132 | 0.0 |
| 133 | 0.0 |
| 134 | 0.0 |
| 135 | 0.0 |
| 136 | 0.0 |
| 137 | 0.0 |
| 138 | 4646.4609375 |
| 139 | 506.832855224609 |
| 140 | 0.0 |
| 141 | 0.0 |
| 142 | 0.0 |
| 143 | 0.0 |
| 144 | 0.0 |
| 145 | 0.0 |83
81
138
81
83
82
82
81
83
m/z
m/z
m/z
m/z
Extended Data Figure 5: Plausible production mechanisms and chemical structures of d-labeled VOLs (a,d, g) and their MS spectra observed in the d-labeled PUFA (b-c, e-f; FA20:4-d8, h-i; FA22:6-d5 +AAPH/hemin peroxidation system.
S8

## Slide 9
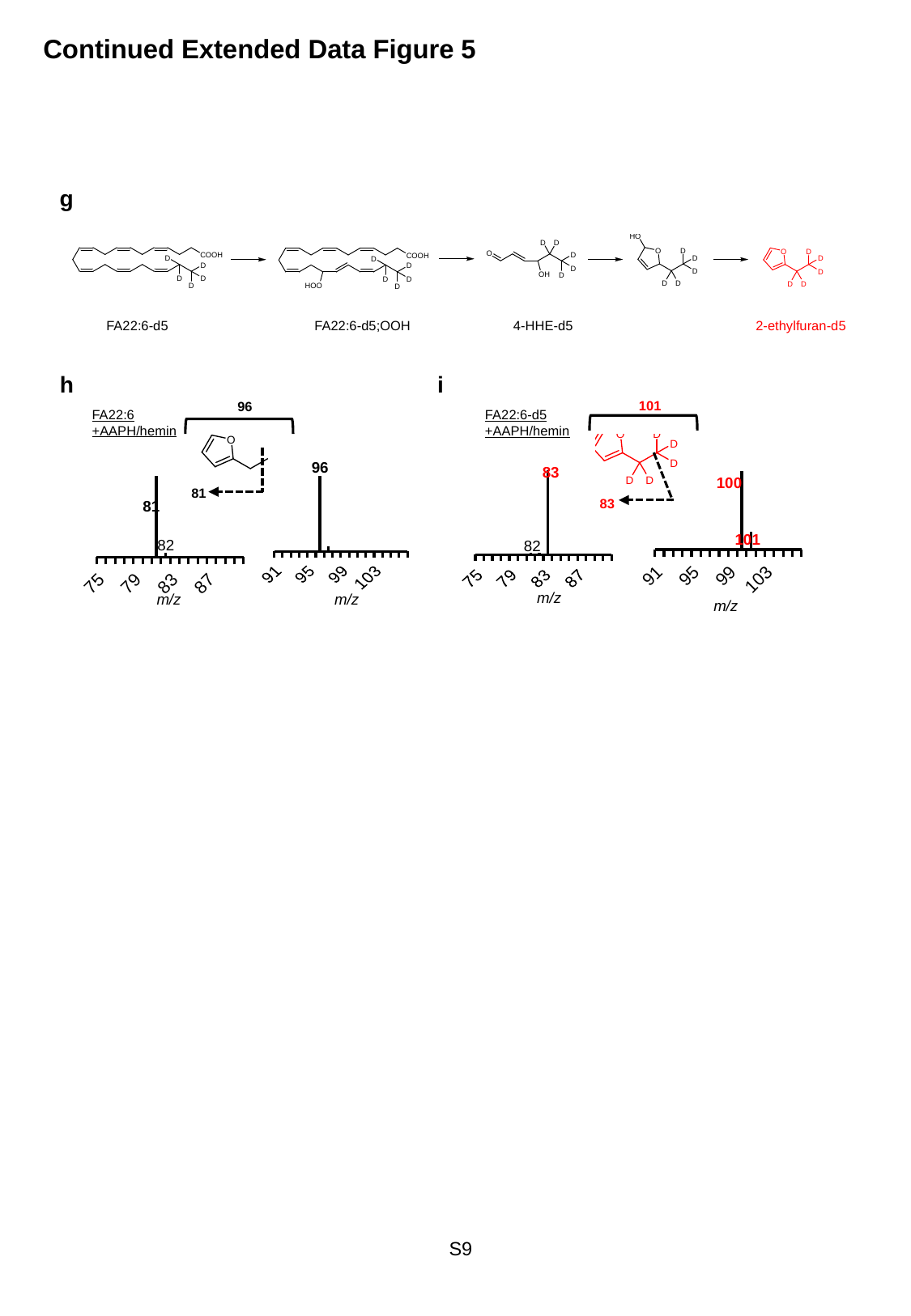

Continued Extended Data Figure 5
g
4-HHE-d5
2-ethylfuran-d5
FA22:6-d5
FA22:6-d5;OOH
h
i
101
96
FA22:6
+AAPH/hemin
FA22:6-d5
+AAPH/hemin
96
### Chart
| Category | |
|---|---|
| 75 | 0.0 |
| 76 | 0.0 |
| 77 | 0.0 |
| 78 | 0.0 |
| 79 | 0.0 |
| 80 | 0.0 |
| 81 | 360.962188720703 |
| 82 | 322.611907958984 |
| 83 | 16656.4765625 |
| 84 | 0.0 |
| 85 | 0.0 |
| 86 | 0.0 |
| 87 | 0.0 |
| 88 | 0.0 |
| 89 | 0.0 |
| 90 | 0.0 |
### Chart
| Category | |
|---|---|
| 91 | 0.0 |
| 92 | 0.0 |
| 93 | 0.0 |
| 94 | 0.0 |
| 95 | 0.0 |
| 96 | 17148.62109375 |
| 97 | 1195.1962890625 |
| 98 | 132.348083496094 |
| 99 | 53.8231887817383 |
| 100 | 260.528778076172 |
| 101 | 64.428337097168 |
| 102 | 0.0 |
| 103 | 0.0 |
| 104 | 0.0 |
| 105 | 0.0 |
| 106 | 0.0 |83
### Chart
| Category | |
|---|---|
| 75 | 0.0 |
| 76 | 0.0 |
| 77 | 0.0 |
| 78 | 0.0 |
| 79 | 0.0 |
| 80 | 0.0 |
| 81 | 52695.7890625 |
| 82 | 2990.5556640625 |
| 83 | 686.128112792969 |
| 84 | 0.0 |
| 85 | 0.0 |
| 86 | 0.0 |
| 87 | 0.0 |
| 88 | 0.0 |
| 89 | 0.0 |
| 90 | 0.0 |
### Chart
| Category | |
|---|---|
| 91 | 0.0 |
| 92 | 0.0 |
| 93 | 0.0 |
| 94 | 0.0 |
| 95 | 0.0 |
| 96 | 131.896743774414 |
| 97 | 152.510345458984 |
| 98 | 637.786987304688 |
| 99 | 584.185302734375 |
| 100 | 32637.853515625 |
| 101 | 7437.7490234375 |
| 102 | 0.0 |
| 103 | 0.0 |
| 104 | 0.0 |
| 105 | 0.0 |
| 106 | 0.0 |100
81
83
81
101
82
82
m/z
m/z
m/z
m/z
S9

## Slide 10
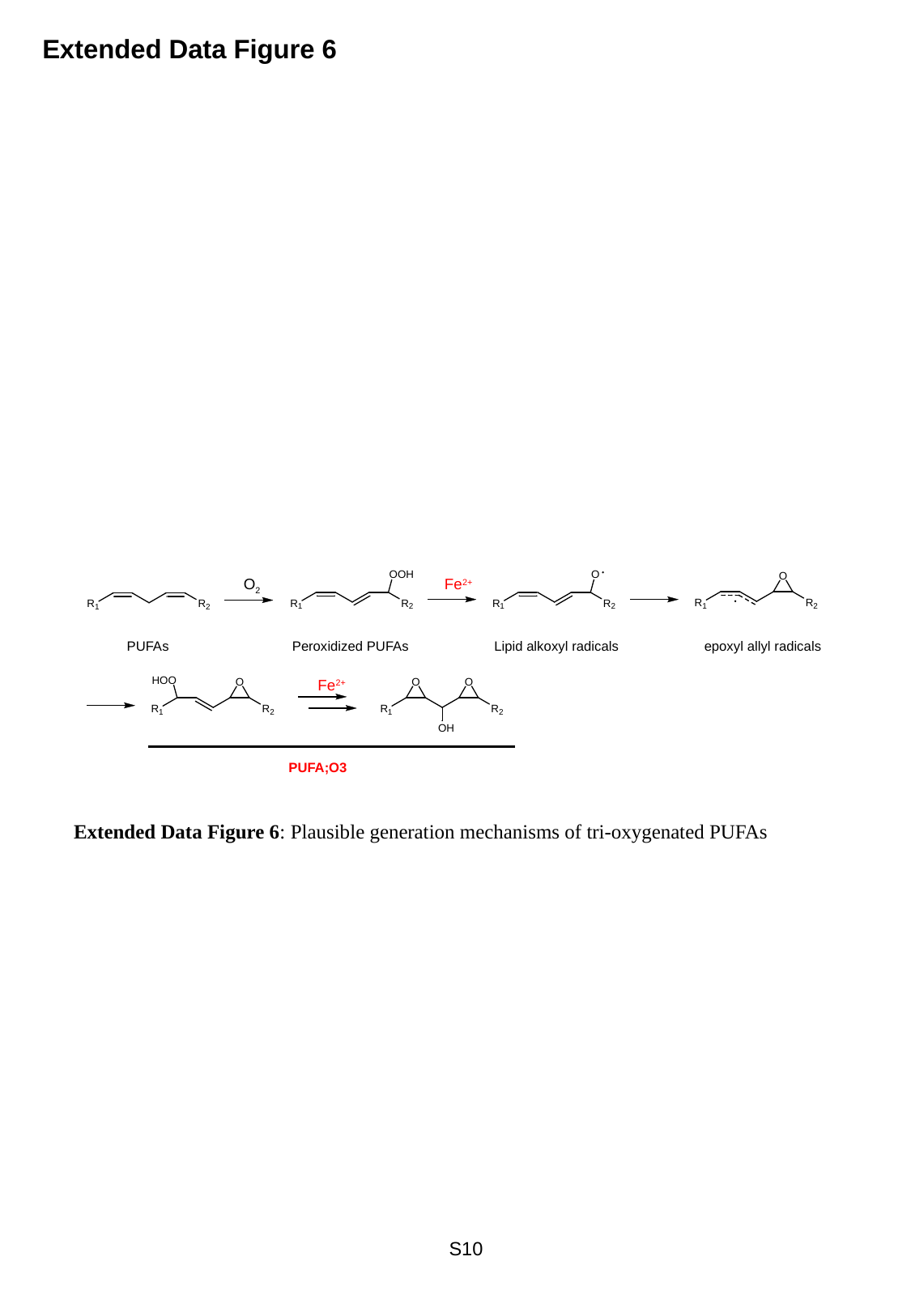

Extended Data Figure 6
O2
Fe2+
epoxyl allyl radicals
Lipid alkoxyl radicals
PUFAs
Peroxidized PUFAs
Fe2+
PUFA;O3
Extended Data Figure 6: Plausible generation mechanisms of tri-oxygenated PUFAs
S10

## Slide 11
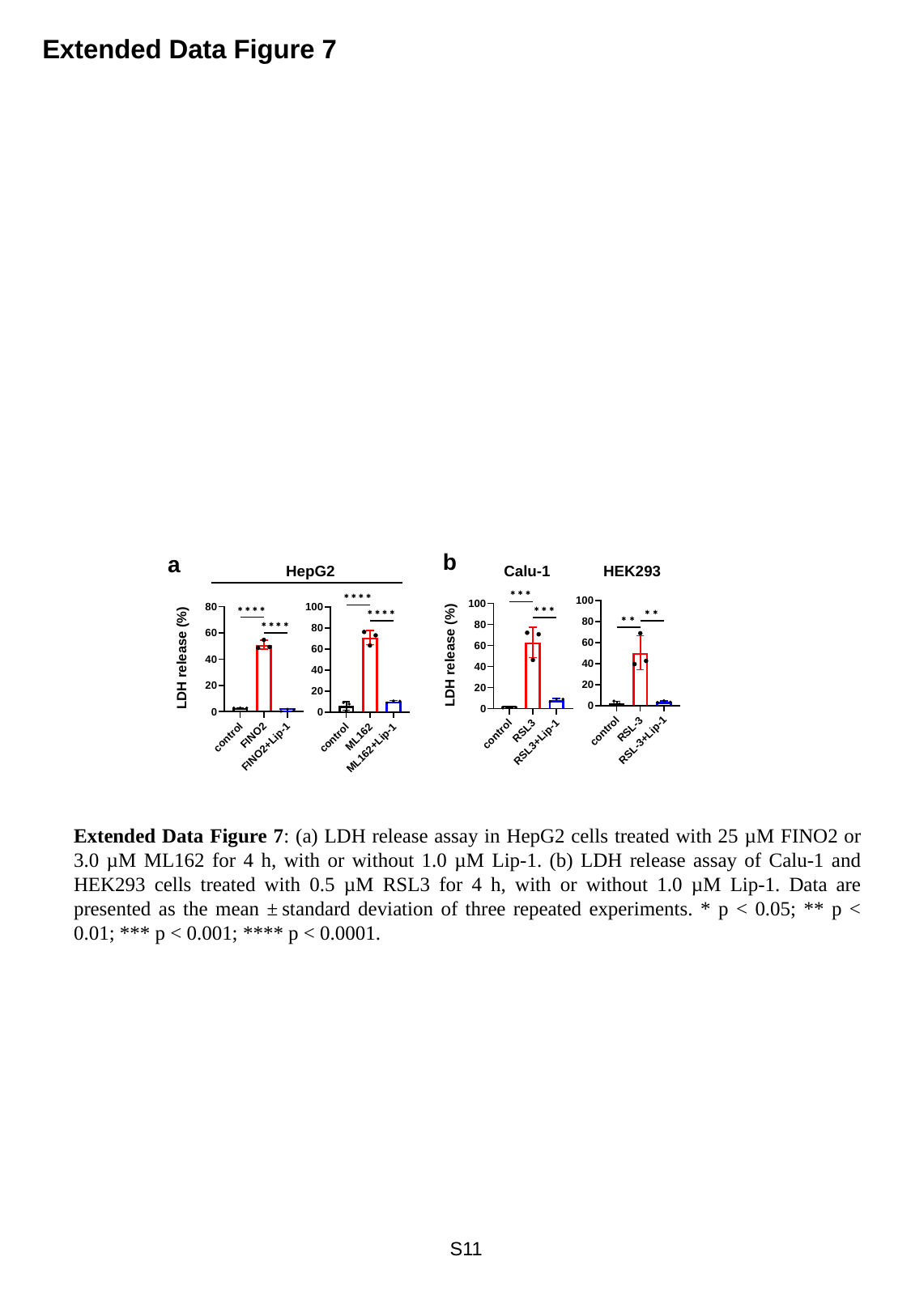

Extended Data Figure 7
b
a
Calu-1
HEK293
HepG2
LDH release (%)
LDH release (%)
Extended Data Figure 7: (a) LDH release assay in HepG2 cells treated with 25 µM FINO2 or 3.0 µM ML162 for 4 h, with or without 1.0 µM Lip-1. (b) LDH release assay of Calu-1 and HEK293 cells treated with 0.5 µM RSL3 for 4 h, with or without 1.0 µM Lip-1. Data are presented as the mean ± standard deviation of three repeated experiments. * p < 0.05; ** p < 0.01; *** p < 0.001; **** p < 0.0001.
S11

## Slide 12
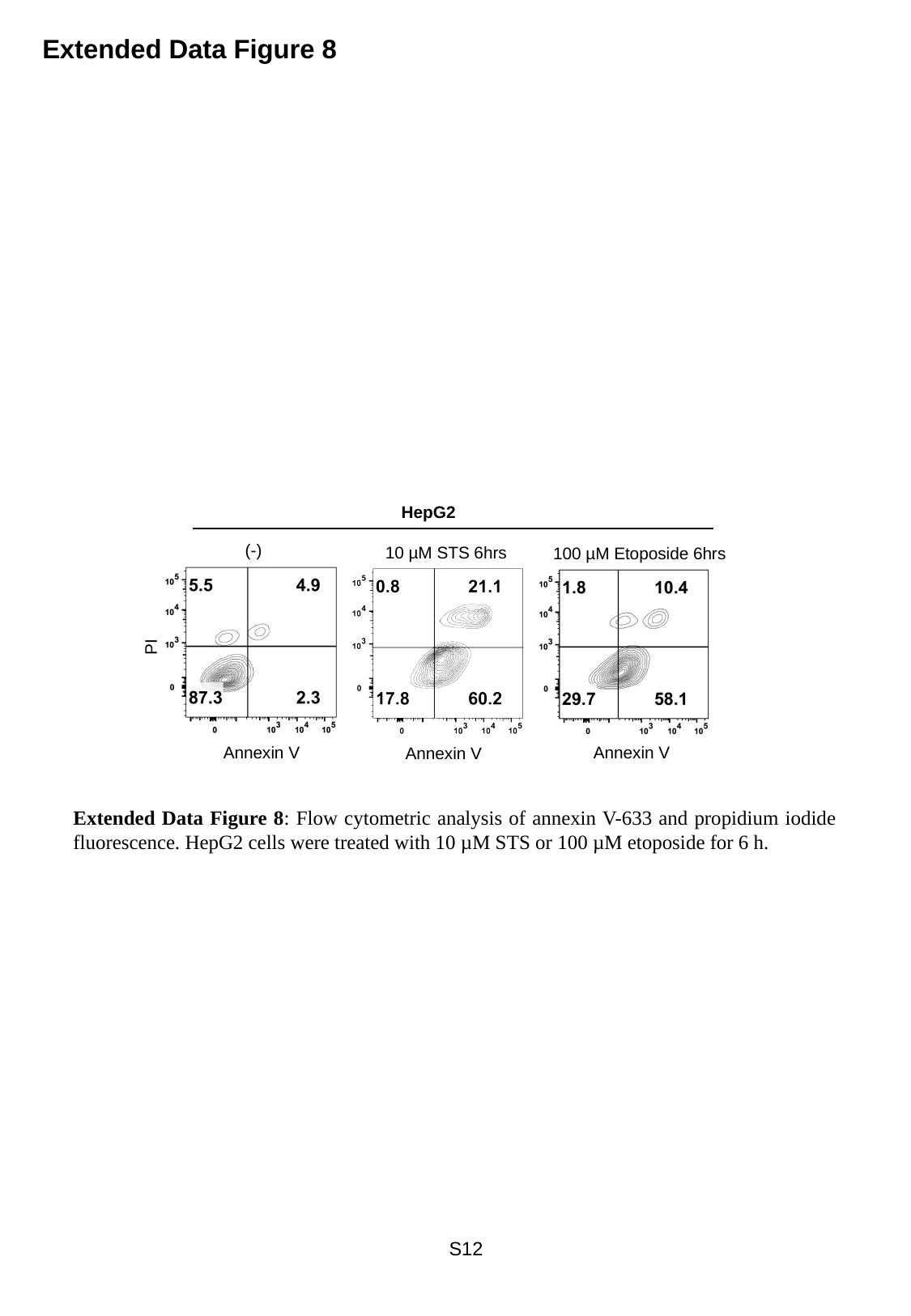

Extended Data Figure 8
HepG2
(-)
10 µM STS 6hrs
100 µM Etoposide 6hrs
PI
Annexin V
Annexin V
Annexin V
Extended Data Figure 8: Flow cytometric analysis of annexin V-633 and propidium iodide fluorescence. HepG2 cells were treated with 10 µM STS or 100 µM etoposide for 6 h.
S12

## Slide 13
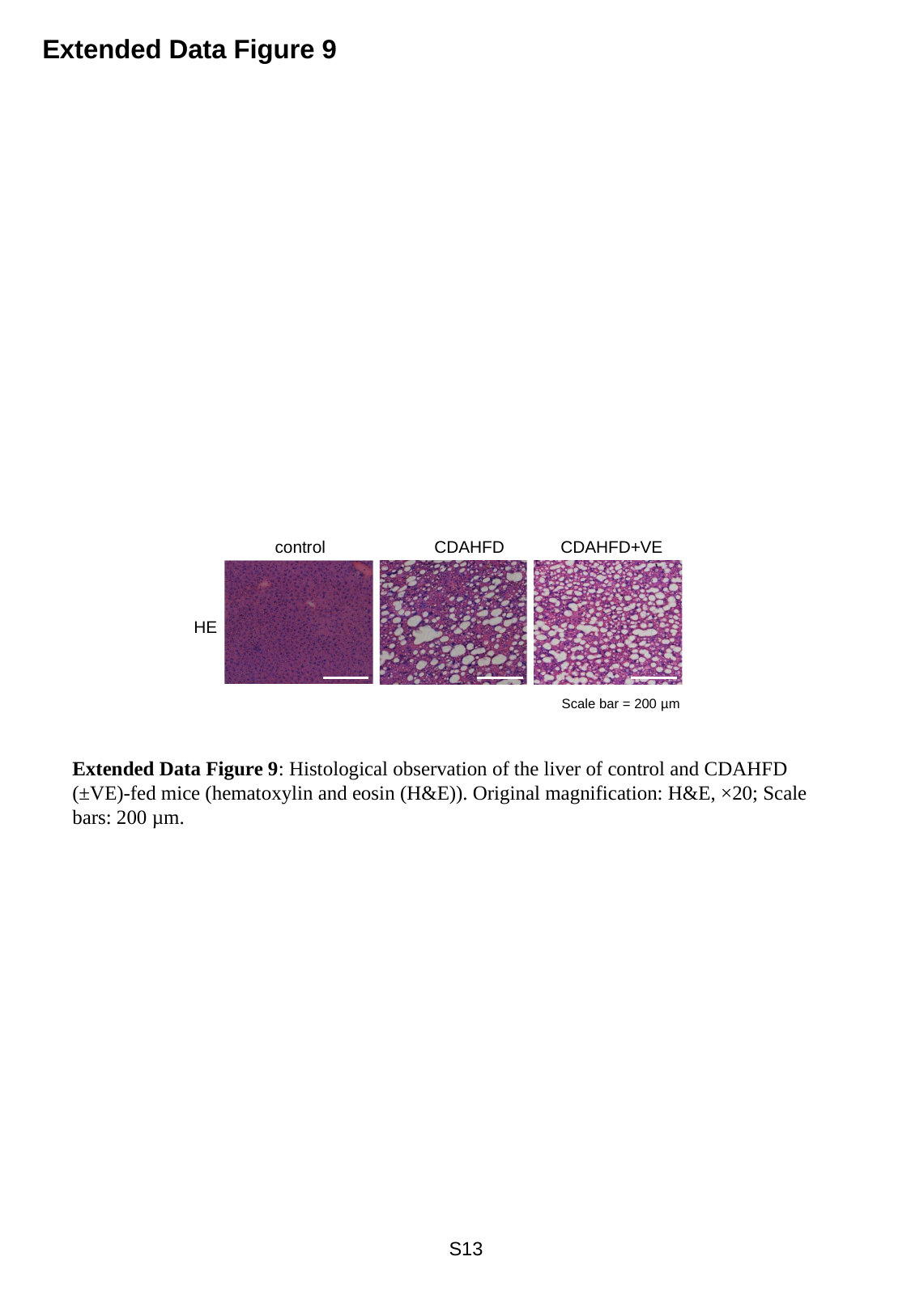

Extended Data Figure 9
control
CDAHFD
CDAHFD+VE
HE
Scale bar = 200 µm
Extended Data Figure 9: Histological observation of the liver of control and CDAHFD (±VE)-fed mice (hematoxylin and eosin (H&E)). Original magnification: H&E, ×20; Scale bars: 200 µm.
S13

## Slide 14
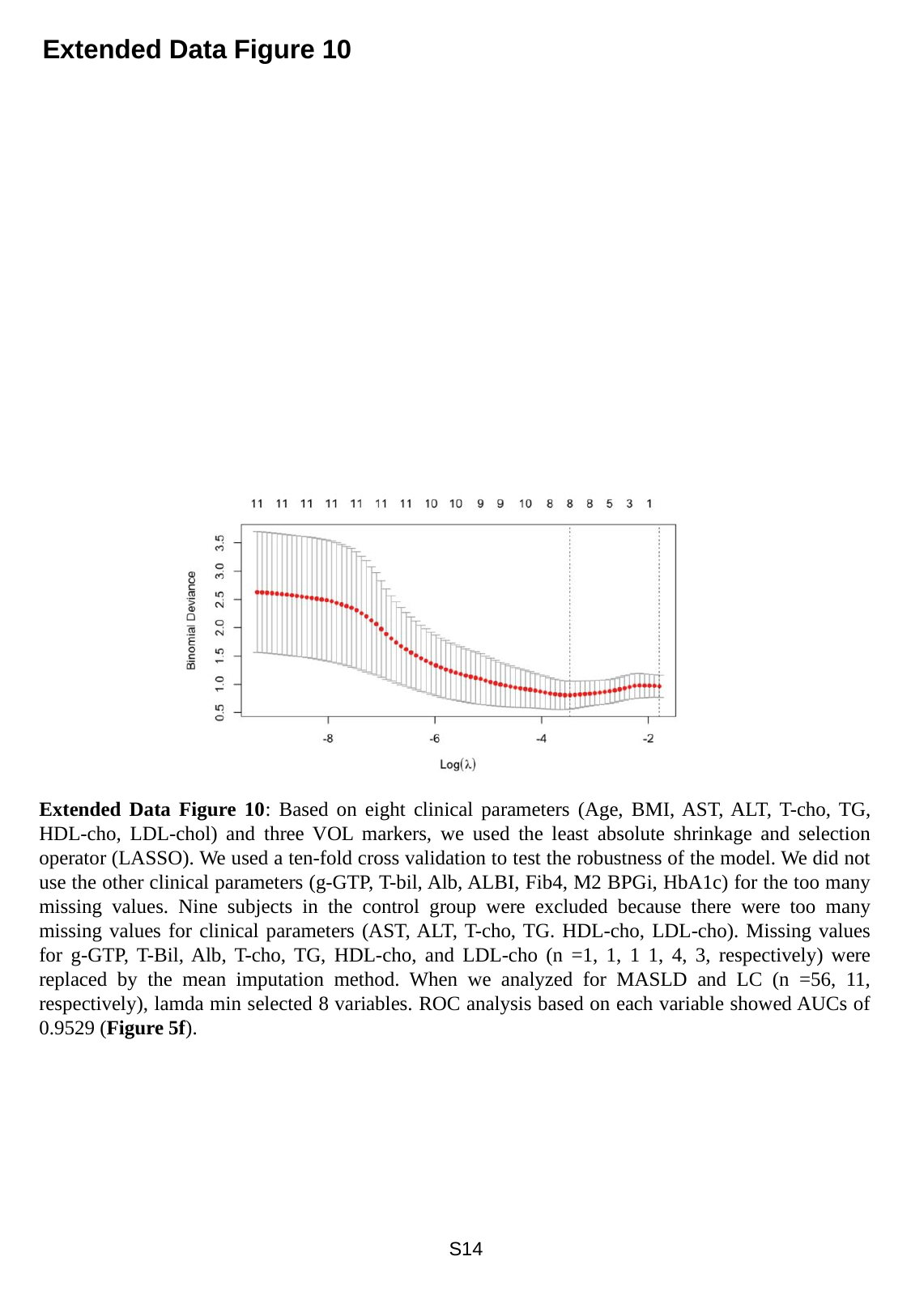

Extended Data Figure 10
Extended Data Figure 10: Based on eight clinical parameters (Age, BMI, AST, ALT, T-cho, TG, HDL-cho, LDL-chol) and three VOL markers, we used the least absolute shrinkage and selection operator (LASSO). We used a ten-fold cross validation to test the robustness of the model. We did not use the other clinical parameters (g-GTP, T-bil, Alb, ALBI, Fib4, M2 BPGi, HbA1c) for the too many missing values. Nine subjects in the control group were excluded because there were too many missing values for clinical parameters (AST, ALT, T-cho, TG. HDL-cho, LDL-cho). Missing values for g-GTP, T-Bil, Alb, T-cho, TG, HDL-cho, and LDL-cho (n =1, 1, 1 1, 4, 3, respectively) were replaced by the mean imputation method. When we analyzed for MASLD and LC (n =56, 11, respectively), lamda min selected 8 variables. ROC analysis based on each variable showed AUCs of 0.9529 (Figure 5f).
S14

## Slide 15
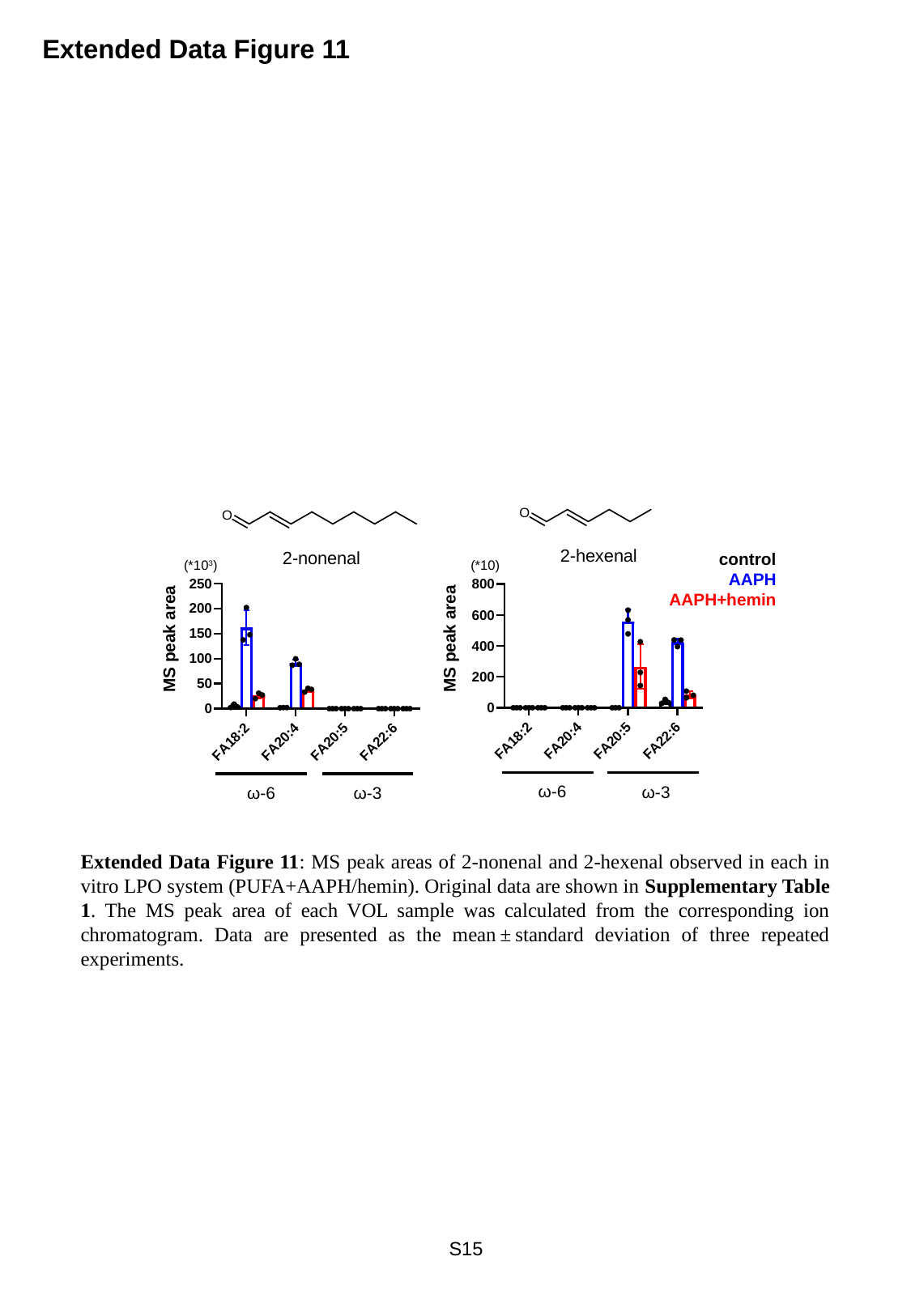

Extended Data Figure 11
2-hexenal
2-nonenal
control
AAPH
AAPH+hemin
(*103)
(*10)
MS peak area
MS peak area
ω-6
ω-3
ω-6
ω-3
Extended Data Figure 11: MS peak areas of 2-nonenal and 2-hexenal observed in each in vitro LPO system (PUFA+AAPH/hemin). Original data are shown in Supplementary Table 1. The MS peak area of each VOL sample was calculated from the corresponding ion chromatogram. Data are presented as the mean ± standard deviation of three repeated experiments.
S15

## Slide 16
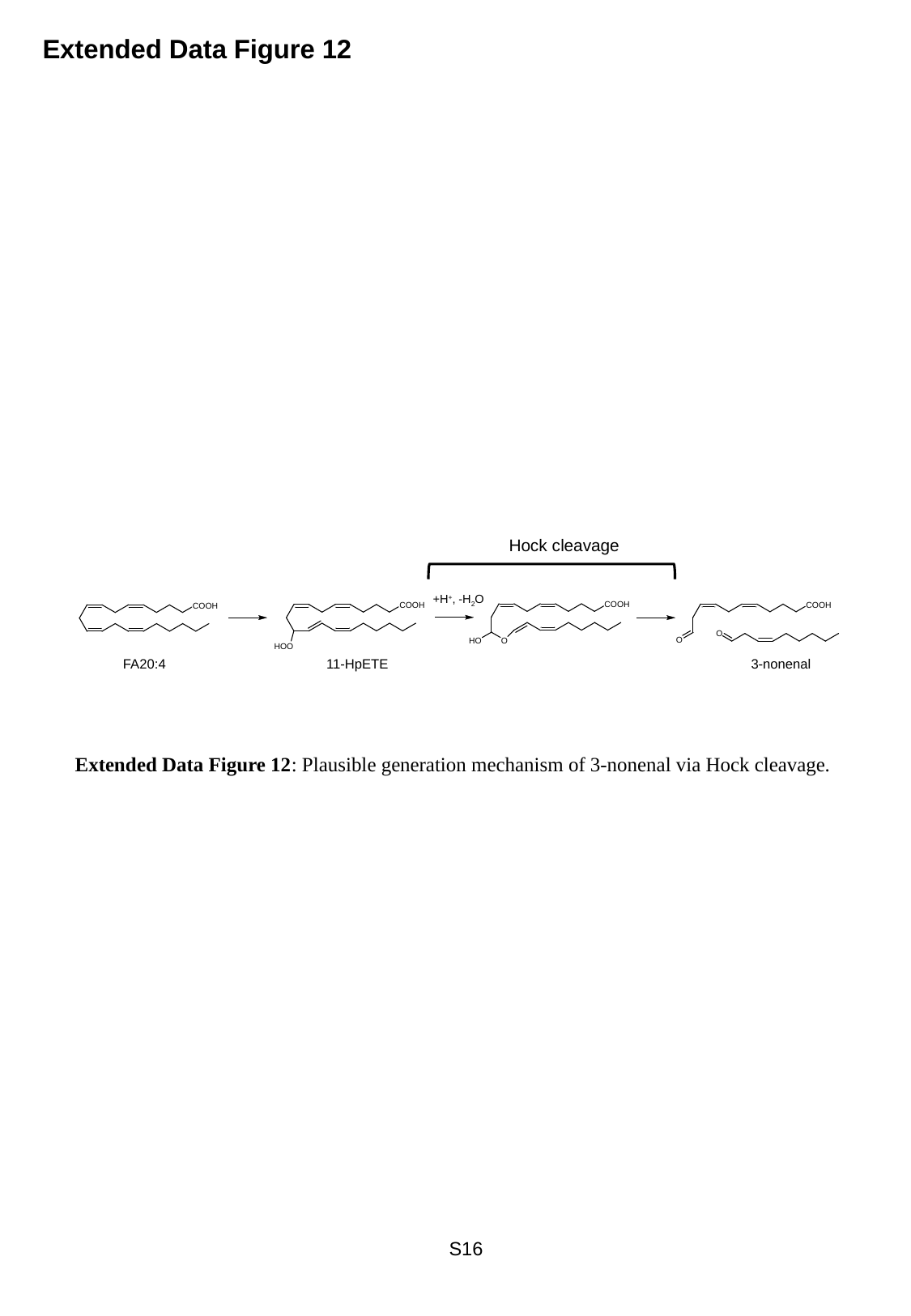

Extended Data Figure 12
Hock cleavage
+H+, -H2O
FA20:4
11-HpETE
3-nonenal
Extended Data Figure 12: Plausible generation mechanism of 3-nonenal via Hock cleavage.
S16

## Slide 17
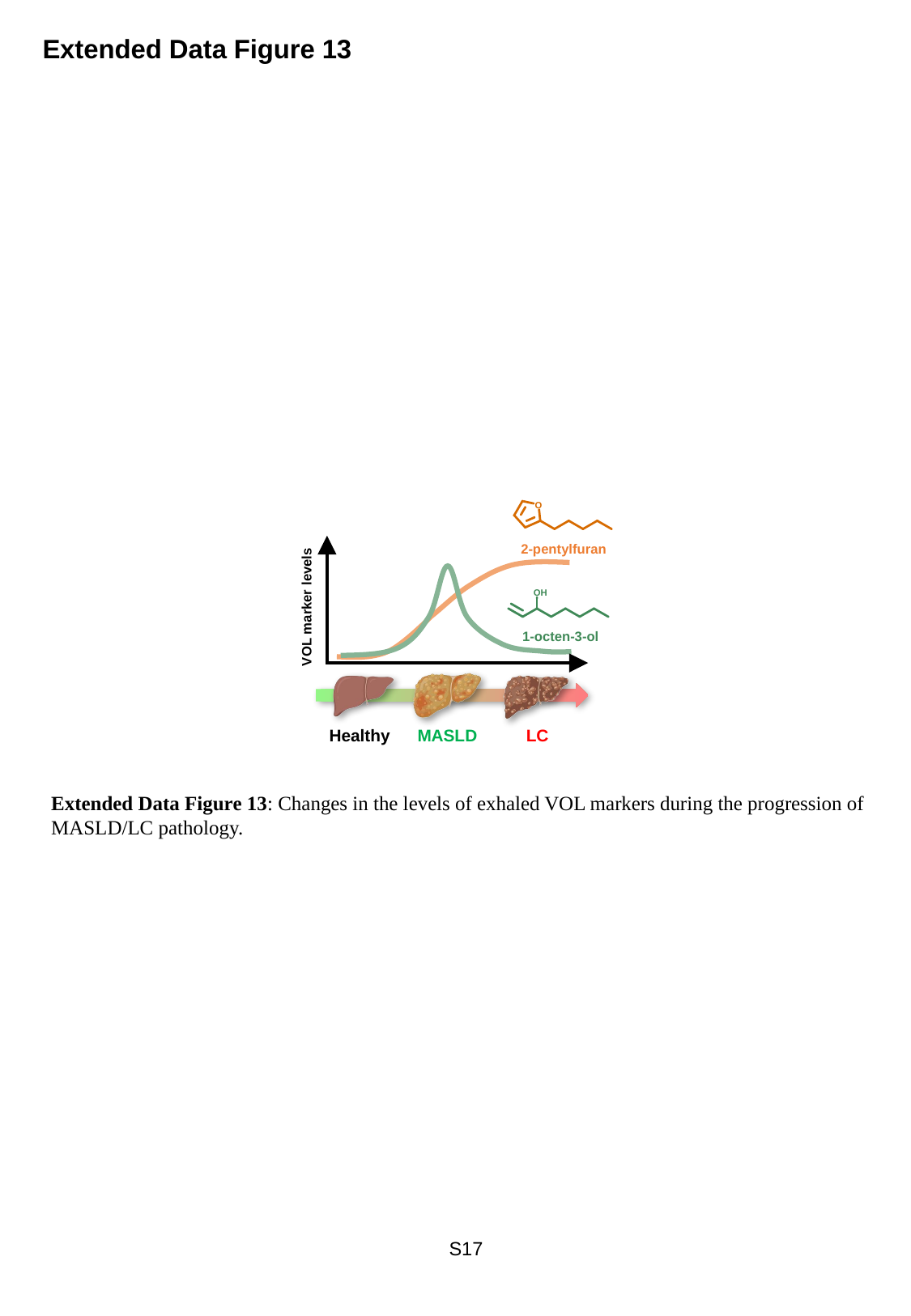

Extended Data Figure 13
2-pentylfuran
VOL marker levels
1-octen-3-ol
Healthy
LC
MASLD
Extended Data Figure 13: Changes in the levels of exhaled VOL markers during the progression of
MASLD/LC pathology.
S17

## Slide 18
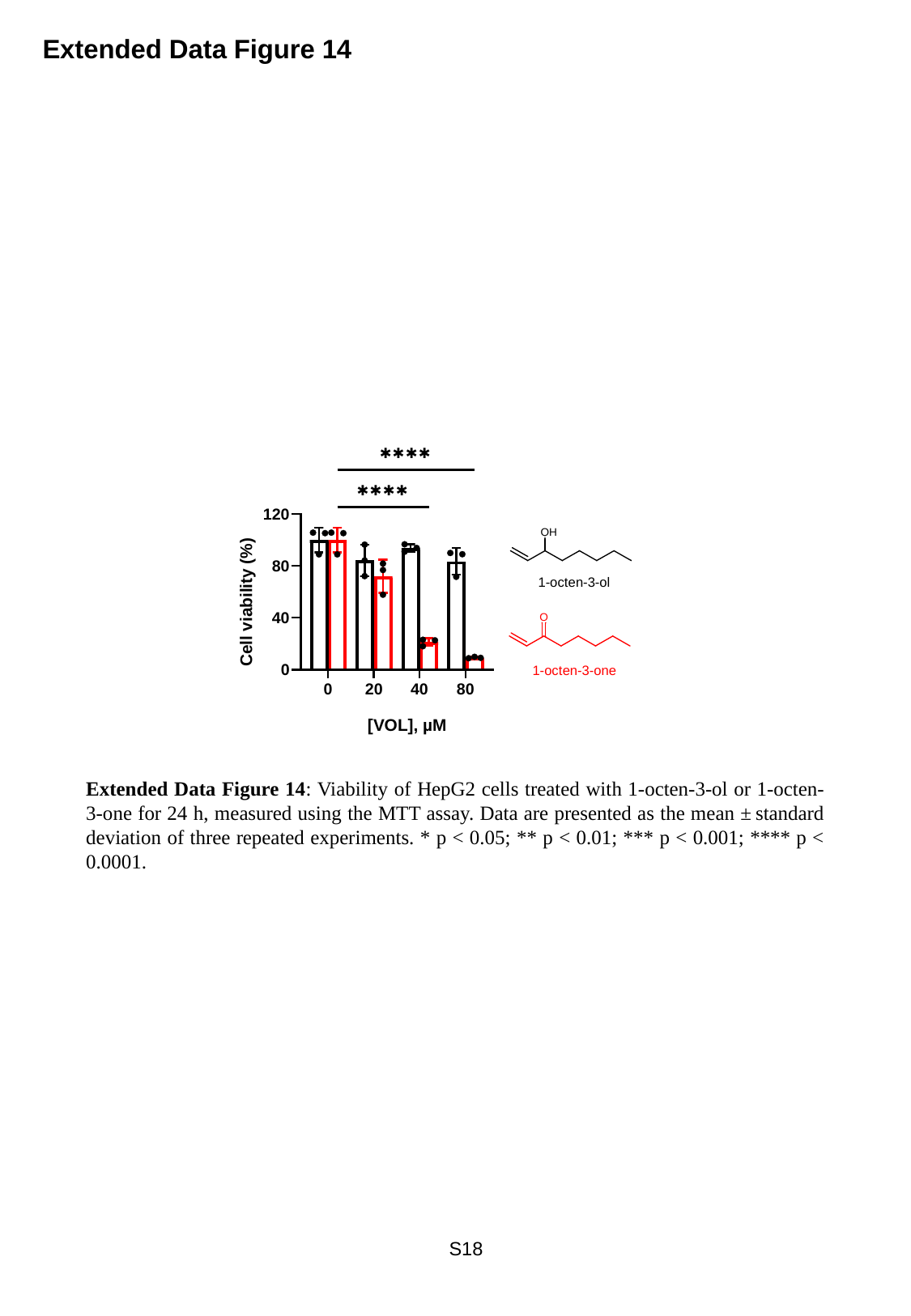

Extended Data Figure 14
1-octen-3-ol
1-octen-3-one
Cell viability (%)
[VOL], µM
Extended Data Figure 14: Viability of HepG2 cells treated with 1-octen-3-ol or 1-octen-3-one for 24 h, measured using the MTT assay. Data are presented as the mean ± standard deviation of three repeated experiments. * p < 0.05; ** p < 0.01; *** p < 0.001; **** p < 0.0001.
S18

## Slide 19
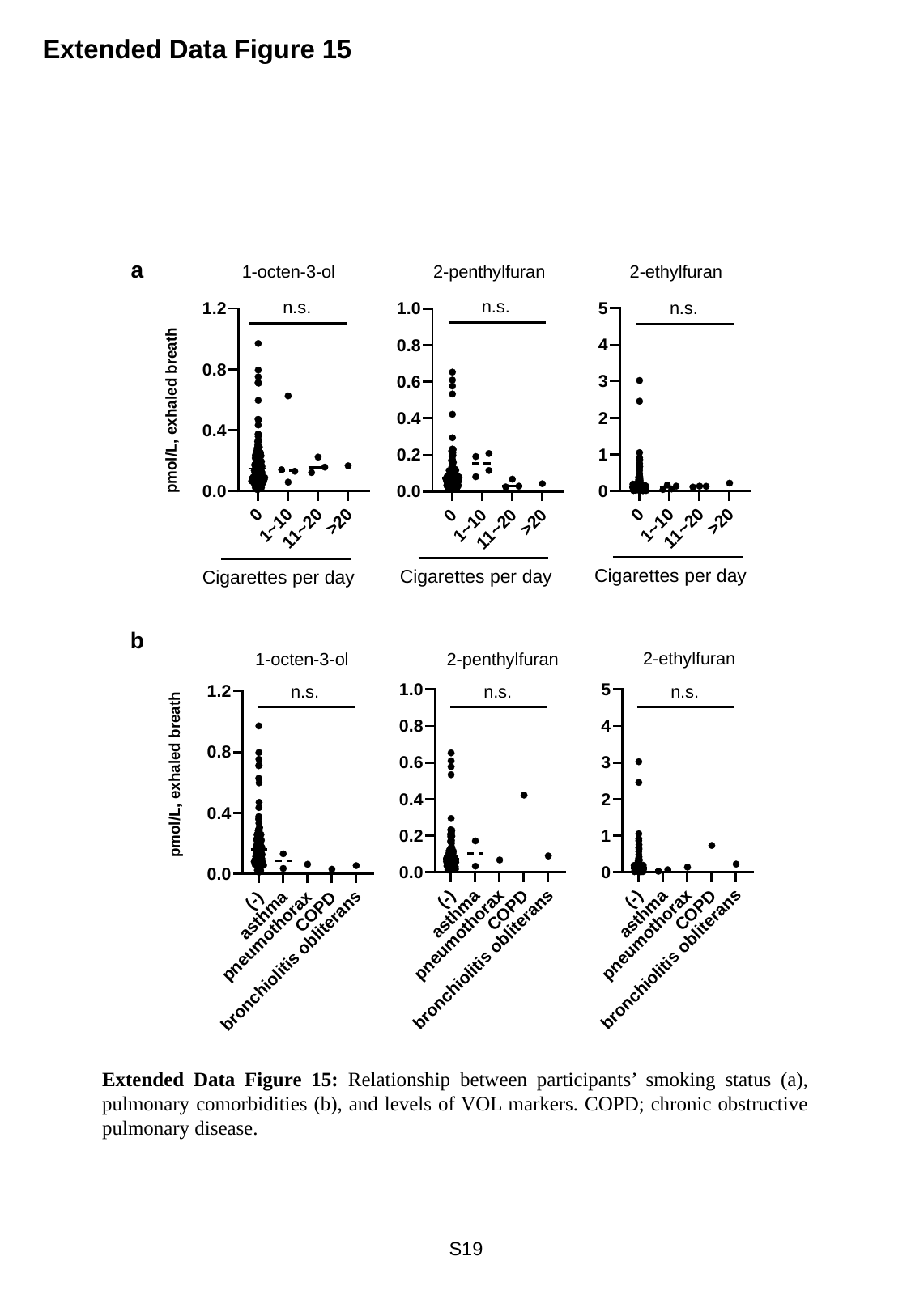

Extended Data Figure 15
a
2-ethylfuran
1-octen-3-ol
2-penthylfuran
n.s.
n.s.
n.s.
pmol/L, exhaled breath
Cigarettes per day
Cigarettes per day
Cigarettes per day
b
2-ethylfuran
1-octen-3-ol
2-penthylfuran
n.s.
n.s.
n.s.
pmol/L, exhaled breath
Extended Data Figure 15: Relationship between participants’ smoking status (a), pulmonary comorbidities (b), and levels of VOL markers. COPD; chronic obstructive pulmonary disease.
S19
